# On the Use of the Lasso for Instrumental Variables Estimation with Some Invalid Instruments

**DOI:** 10.1080/01621459.2018.1498346

**Published:** 2018-11-13

**Authors:** Frank Windmeijer, Helmut Farbmacher, Neil Davies, George Davey Smith

**Affiliations:** a Department of Economics, University of Bristol, Bristol, United Kingdom; b MRC Integrative Epidemiology Unit, Bristol, United Kingdom; c Center for the Economics of Aging, Max Planck Society Munich, Germany; d School of Social and Community Medicine, University of Bristol, Bristol, United Kingdom

**Keywords:** Causal inference, Instrumental variables estimation, Invalid instruments, Lasso, Mendelian randomization.

## Abstract

We investigate the behavior of the Lasso for selecting invalid instruments in linear instrumental variables models for estimating causal effects of exposures on outcomes, as proposed recently by Kang et al. Invalid instruments are such that they fail the exclusion restriction and enter the model as explanatory variables. We show that for this setup, the Lasso may not consistently select the invalid instruments if these are relatively strong. We propose a median estimator that is consistent when less than 50% of the instruments are invalid, and its consistency does not depend on the relative strength of the instruments, or their correlation structure. We show that this estimator can be used for adaptive Lasso estimation, with the resulting estimator having oracle properties. The methods are applied to a Mendelian randomization study to estimate the causal effect of body mass index (BMI) on diastolic blood pressure, using data on individuals from the UK Biobank, with 96 single nucleotide polymorphisms as potential instruments for BMI. Supplementary materials for this article are available online.

## 1. Introduction

Instrumental variables estimation is a procedure for the identification and estimation of causal effects of exposures on outcomes where the observed relationships are confounded by nonrandom selection of exposure. This problem is likely to occur in observational studies, but also in randomized clinical trials if there is selective participant noncompliance. An instrumental variable (IV) can be used to solve the problem of nonignorable selection. To do this, an IV needs to be associated with the exposure, but only associated with the outcome indirectly through its association with the exposure. The former condition is referred to as the “relevance” and the latter as the “exclusion” condition. Examples of instrumental variables are quarter-of-birth for educational achievement to determine its effect on wages, see Angrist and Krueger (1991), randomization of patients to treatment as an instrument for actual treatment when there is noncompliance, see, for example, Greenland (2000), and Mendelian randomization studies use IVs based on genetic information, see, for example, Lawlor et al. (2008). For recent reviews and further examples see, for example, Clarke and Windmeijer (2012), Imbens (2014), Burgess, Small, and Thompson (2017), and Kang et al. (2016).

Whether instruments are relevant can be tested from the observed association between exposure and instruments. The effects on the standard linear IV estimator of “weak instruments,” that is, the case where instruments are only weakly associated with the exposure of interest, have been derived for the linear model using weak instrument asymptotics by Staiger and Stock (1997). This has led to the derivation of critical values for the simple *F*-test statistic for testing the null of weak instruments by Stock and Yogo (2005). Another strand of the literature focuses on instrument selection in potentially high-dimensional settings, see, for example, Belloni et al. (2012), Belloni et al. (2014), Chernozhukov et al. (2015), and Lin et al. (2015), where the focus is on identifying important covariate effects and selecting optimal instruments from a (large) set of a priori valid instruments, where optimality is with respect to the variance of the IV estimator.

In this article, we consider violations of the exclusion condition of the instruments, following closely the setup by Kang et al. (2016) for the linear IV model where some of the available instruments can be invalid in the sense that they can have a direct effect on the outcomes or are associated with unobserved confounders. Kang et al. (2016) proposed a Lasso-type procedure to identify and select the set of invalid instruments. Liao (2013) and Cheng and Liao (2015) also considered shrinkage estimation for identification of invalid instruments, but in their setup there is a subset of instruments that is known to be valid and that contains sufficient information for identification and estimation of the causal effects. In contrast, Kang et al. (2016) did not assume any prior knowledge about which instruments are potentially valid or invalid. This is a similar setup as in Andrews (1999) who proposed a selection procedure using information criteria based on the so-called *J*-test of over-identifying restrictions, as developed by Sargan (1958) and Hansen (1982). The Andrews (1999) setup is more general than that of Kang et al. (2016) and requires a large number of model evaluations, which has a negative impact on the performance of the selection procedure.

This article assesses the performance of the Kang et al. (2016) Lasso-type selection and estimation procedure in their setting of a fixed number of potential instruments. If the set of invalid instruments were known, the oracle two-stage least squares (2SLS) estimator would be the estimator of choice in their setting. As the focus is estimation of and inference on the causal effect parameter, denoted by β, and as the standard Lasso approach does not have oracle properties, see, for example, Zou (2006), we show how the adaptive Lasso procedure by Zou (2006) can be used to obtain an estimator with oracle properties. To do so, we propose an initial consistent estimator of the parameters that is consistent also when the irrepresentable condition for consistent Lasso selection of Zhao and Yu (2006) and Zou (2006) fails. The oracle property in this setup is when an estimator for β has the same limiting distribution as the oracle 2SLS estimator.

Applying the irrepresentable condition to this IV setup, we derive conditions under which the Lasso method does not consistently select the invalid instruments. As is well known from Zhao and Yu (2006), Zou (2006), Meinshausen and Bühlmann (2006), and Wainwright (2009), certain correlation structures of the variables prevent consistent selection. New in our results are the conditions on the strength of the invalid instruments relative to that of the valid ones that result in violations of the irrepresentable condition, where the strength of an instrument is its standardized effect on the exposure. From this we can show that consistent selection of the invalid instruments may not be possible if these are relatively strong, even when less than 50% of the instruments are invalid, which is a sufficient condition for the identification of the parameters.

We show that under the condition that less than 50% of the instruments are invalid, a simple median-type estimator is a consistent estimator for the parameters in the model, independent of the strength of the invalid instruments relative to that of the valid instruments, or their correlation structure. It can therefore be considered for use in the adaptive Lasso procedure as proposed by Zou (2006). With *n* the sample size, we show that the median estimator converges at the $\sqrt{n}$ rate, but with an asymptotic bias, as the limiting distribution is that of an order statistic. It does, however, satisfy the conditions for the adaptive Lasso procedure to enjoy oracle properties.

Because of this oracle property, and as in practice instrument strength is very likely to vary by instruments and invalid instruments could be relatively strong, it will be important to consider our adaptive Lasso approach for assessing instrument validity and estimating causal effects. In Mendelian randomization studies it is clear that genetic markers have differential impacts on exposures from examining the results from genome-wide association studies and one cannot rule out ex ante that invalid instruments with a direct effect are also stronger predictors for the exposure. (Bowden et al. (2015) and Kolesar et al. (2015) allowed for all instruments to be invalid and showed that the causal effect can be consistently estimated if the number of instruments increases with the sample size under the assumption of uncorrelatedness of the instrument strength and their direct effects on the outcome variable.)

The next section, Section 2, introduces the model and the Lasso estimator as proposed by Kang et al. (2016). In Section 3, we derive the irrepresentable condition for this particular Lasso selection problem and present the result on the relationship between the relative strengths of the instruments and consistent selection. Section 4 presents the median estimator, establishes its consistency, and shows that its asymptotic properties are such that the adaptive Lasso estimator enjoys oracle properties. Section 5 presents some Monte Carlo simulation results. In Section 5.2, we link the Andrews (1999) method to the Lasso selection problem and show how the test of overidentifying restrictions can be used as a stopping rule. Section 5.3 investigates how close the behavior of the adaptive Lasso estimator is to that of the oracle 2SLS estimator in the Monte Carlo simulations, by comparing the performances of the Wald tests on the causal parameter under the null for different sample sizes. Further analyses and simulation results investigating the effects of varying the information content by varying the strength of the instruments and the size of the direct effects of the invalid instruments on the outcome are presented in Section B in the supplementary materials. In Section 6, the methods are applied to a Mendelian randomization study to estimate the causal effect of body mass index (BMI) on diastolic blood pressure using data on individuals from the UK Biobank, with 96 single nucleotide polymorphisms as potential instruments for BMI. Section 7 concludes.

The following notation is used in the remainder of the article. For a full column rank matrix **X** with *n* rows, **M**_*X*_ = **I**_*n*_ − **P**_*X*_, where **P**_*X*_ = **X**(**X**′**X**)^− 1^**X**′ is the projection onto the column space of **X**, and **I**_*n*_ is the *n* -dimensional identity matrix. A *k*-vector of ones is denoted as ***ι***_*k*_. The *l_p_*-norm is denoted by ‖.‖_*p*_, and the *l*_0_-norm, ‖.‖_0_, denotes the number of nonzero components of a vector. We use ‖.‖_∞_ to denote the maximal element of a vector.

## 2. Model and Lasso Estimator

We follow Kang et al. (2016; KZCS from now on), who considered the following potential outcomes model. For *i* = 1, …, *n*, let *Y*^(*d*, **z**)^_*i*_, be the potential outcome if the individual *i* were to have exposure *d* and instrument values **z**. The observed outcome for an individual *i* is denoted by the scalar *Y_i_*, the treatment by the scalar *D_i_*, and the vector of *L* potential instruments by **Z**_*i*._. The instruments may not all be valid and can have a direct or indirect effect. For two possible values of the exposure d*, *d* and instruments z*, **z** , assume the following potential outcomes model

(1) $$Y_{i}^{\left(d^{*},z^{*}\right)}-Y_{i}^{\left(d,z\right)}={\left(z^{*}-z\right)}^{'}\phi +\left(d^{*}-d\right)\beta$$

(2) $$E[Y_{i}^{\left(0,0\right)}|Z_{i.}]=Z_{i.}^{'}\psi ,$$

where ***φ*** measures the direct effect of **z** on *Y*, and ***ψ*** represents the presence of unmeasured confounders that affect both the instruments and the outcome.

We have a random sample {*Y_i_*, *D_i_*, **Z**′_*i*._}^*n*^_*i* = 1_. Combining (1) and (2), the observed data model for the random sample is given by

(3) $$Y_{i}=D_{i}\beta +Z_{i.}^{'}\alpha +\varepsilon_{i},$$

where ***α*** = ***φ*** + ***ψ***;

$$\varepsilon_{i}=Y_{i}^{\left(0,0\right)}-E[Y_{i}^{\left(0,0\right)}|Z_{i.}]$$

and hence *E*[ϵ_*i*_|**Z**_*i*._] = 0. For ease of exposition, we further assume that *E*[ϵ^2^_*i*_|**Z**_*i*._] = σ^2^_ϵ_.

The KZCS definition of a valid instrument is then linked to the exclusion restriction and given as follows: Instrument *j*, *j* ∈ {1, …, *L*}, is valid if α_*j*_ = 0 and it is invalid if α_*j*_ ≠ 0. As in the KZCS setting, we are interested in the identification and estimation of the scalar treatment effect β in large samples with a fixed number *L* of potential instruments.

Let **y** and **d** be the *n*-vectors of *n* observations on {*Y_i_*} and {*D_i_*}, respectively, and let **Z** be the *n* × *L* matrix of potential instruments. As an intercept is implicitly present in the model, **y**, **d**, and the columns of **Z** have all been taken in deviation from their sample means. Following the notation of Zou (2006), let **Z**_*A*_ be the set of invalid instruments, *A* = {*j*: α_*j*_ ≠ 0} and ***α***_*A*_ the associated coefficient vector. The oracle instrumental variables or two-stage least square (2SLS) estimator is obtained when the set **Z**_*A*_ is known. Let $R_{A}=\left[\begin{array}{cc} d & Z_{A} \end{array}\right]$, the oracle 2SLS estimator is then given by

(4) $${\hat{\theta }}_{\text{or}}=\left(\begin{array}{c} {\hat{\beta }}_{\text{or}}\\ {\hat{\alpha }}_{A} \end{array}\right)={\left(R_{A}^{'}P_{Z}R_{A}\right)}^{-1}R_{A}^{'}P_{Z}y.$$

Let $\hat{d}=P_{Z}d$, with individual elements ${\hat{D}}_{i}$, then ${\hat{\theta }}_{\text{or}}$ is the OLS estimator in the model

$$Y_{i}={\hat{D}}_{i}\beta +Z_{A,i.}^{'}\alpha_{A}+\xi_{i},$$

where ξ_*i*_ is defined implicitly, and hence

(5) $${\hat{\alpha }}_{A}={\left(Z_{A}^{'}M_{\hat{d}}Z_{A}\right)}^{-1}Z_{A}^{'}M_{\hat{d}}y={\left(Z_{A}^{'}M_{\hat{d}}Z_{A}\right)}^{-1}Z_{A}^{'}M_{\hat{d}}P_{Z}y.$$

The oracle 2SLS estimator for β is given by

$${\hat{\beta }}_{\text{or}}={\left({\hat{d}}^{'}M_{Z_{A}}\hat{d}\right)}^{-1}{\hat{d}}^{'}M_{Z_{A}}y.$$

Under standard assumptions, as defined below,

(6) $$\sqrt{n}\left({\hat{\beta }}_{\text{or}}-\beta \right)\overset{d}{⟶}N\left(0,\sigma_{\beta_{\text{or}}}^{2}\right),$$

where

(7) $$\begin{array}{c} \sigma_{\beta_{\text{or}}}^{2}=\sigma_{\varepsilon }^{2}(E{\left[Z_{i.}D_{i}\right]}^{'}E{\left[Z_{i.}Z_{i.}^{'}\right]}^{-1}E\left[Z_{i.}D_{i}\right]\\ -E{\left[Z_{A,i.}D_{i}\right]}^{'}E{\left[Z_{A,i.}Z_{A,i.}^{'}\right]}^{-1}E\left[Z_{A,i.}D_{i}\right]{)}^{-1}. \end{array}$$

The vector $\hat{d}$ is the linear projection of **d** on **Z**. If we define $\hat{\gamma }={\left(Z^{'}Z\right)}^{-1}Z^{'}d$, then $\hat{d}=Z\hat{\gamma }$, or ${\hat{D}}_{i}=Z_{i.}^{'}\hat{\gamma }$. We specify

(8) $$D_{i}=Z_{i.}^{'}\gamma +v_{i},$$

where ***γ*** = *E*[**Z**_*i*._**Z**′_*i*._]^− 1^*E*[**Z**_*i*._*D_i_*], and hence *E*[**Z**_*i*._*v_i_*] = 0. Further, as in KZCS, let ***Γ*** = *E*[**Z**_*i*._**Z**′_*i*._]^− 1^*E*[**Z**_*i*._*Y_i_*] = ***γ***β + ***α***. Then define π_*j*_ as

(9) $$\pi_{j}\equiv \frac{\Gamma_{j}}{\gamma_{j}}=\beta +\frac{\alpha_{j}}{\gamma_{j}},$$

for *j* = 1, …, *L*. Theorem 1 in KZCS states the conditions under which, given knowledge of ***γ*** and ***Γ***, a unique solution exists for values of β and α_*j*_. A necessary and sufficient condition to identify β and the α_*j*_ is that the valid instruments form the largest group, where instruments form a group if they have the same value of π. Corollary 1 in KZCS then states a sufficient condition for identification. Let *s* = ||***α***||_0_ be the number of invalid instruments. A sufficient condition is that *s* < *L*/2, as then clearly the largest group is formed by the valid instruments.

In model (3), some elements of ***α*** are assumed to be zero, but it is not known ex ante which ones they are and the selection problem therefore consists of correctly identifying those instruments with nonzero α. KZCS proposed to estimate the parameters ***α*** and β by using *l*_1_ penalization on ***α*** and to minimize

(10) $$\begin{array}{c} \left({\hat{\alpha }}^{\left(n\right)},{\hat{\beta }}^{\left(n\right)}\right)=\arg\min_{\alpha ,\beta }\frac{1}{2}∥P_{Z}\left(y-d\beta -Z\alpha \right){∥}_{2}^{2}+\lambda_{n}{∥\alpha ∥}_{1}, \end{array}$$

where ‖***α***‖_1_ = ∑_*j*_|α_*j*_|. This method is closely related to the Lasso, and the regularization parameter λ_*n*_ determines the sparsity of the vector ${\hat{\alpha }}^{\left(n\right)}$. From (5), a fast two-step algorithm is proposed as follows. For a given λ_*n*_ solve

(11) $${\hat{\alpha }}^{\left(n\right)}=\arg\min_{\alpha }\frac{1}{2}∥M_{\hat{d}}P_{Z}y-M_{\hat{d}}{Z\alpha ∥}_{2}^{2}+\lambda_{n}{∥\alpha ∥}_{1}$$

and obtain ${\hat{\beta }}^{\left(n\right)}$ by

(12) $${\hat{\beta }}^{\left(n\right)}=\frac{{\hat{d}}^{'}\left(y-Z{\hat{\alpha }}^{\left(n\right)}\right)}{{\hat{d}}^{'}\hat{d}}.$$

To find ${\hat{\alpha }}^{\left(n\right)}$ in (11), the Lasso modification of the LARS algorithm of Efron et al. (2004) can be used and KZCS had developed an R-routine for this purpose, called *sisVIVE* (some invalid and some valid IV estimator), where the regularization parameter λ_*n*_ is obtained by cross-validation.

For the random variables and iid sample {*Y_i_*, *D_i_*, **Z**′_*i*._}^*n*^_*i* = 1_, and model (3) and (8), we assume throughout that the following conditions hold:

Assumption 1. *E*[**Z**_*i*._**Z**′_*i*._] = **Q**, with **Q** a finite and full-rank matrix.

Assumption 2. Let **u**_*i*_ = (ϵ_*i*_*v_i_*)′. Then *E*[**u**_*i*_] = 0; $E\left[u_{i}u_{i}^{'}\right]=[\begin{array}{cc} \sigma_{\varepsilon }^{2} & \sigma_{\varepsilon v}\\ \sigma_{\varepsilon v} & \sigma_{v}^{2} \end{array}]=\Sigma$. The elements of Σ are finite.

Assumption 3. plim(*n*^− 1^**Z**′**Z**) = *E*[**Z**_*i*._**Z**′_*i*._]; plim(*n*^− 1^**Z**′**d**) = *E*[**Z**_*i*._*D_i_*]; plim(*n*^− 1^**Z**′***ϵ***) = *E*[**Z**_*i*._ϵ_*i*_] = 0; plim(*n*^− 1^**Z**′**v**) = *E*[**Z**_*i*._*v_i_*] = 0; plim(*n*^− 1^∑^*n*^_*i* = 1_**u**_*i*_) = 0; plim(*n*^− 1^∑^*n*^_*i* = 1_**u**_*i*_**u**′_*i*_) = Σ.

Assumption 4. γ = (*E*[**Z**_*i*._**Z**′_*i*._])^− 1^*E*[**Z**_*i*._*D_i_*], γ_*j*_ ≠ 0, *j* = 1, …, *L*.

The setting is thus a relatively straightforward one with fixed parameters β, ***α***, and ***γ***, and fixed number *L* ≪ *n* of potential instruments. This is the setting under which the oracle 2SLS estimator has the limiting distribution (6), and is a setting of interest in many applications. To identify in this simple setting an ex ante unknown subset of invalid instruments using the Lasso is challenging, as highlighted in the next section where we investigate the irrepresentable condition for this setting.

For the case of many weak instruments, even the oracle 2SLS estimator would not be the estimator of choice, due to its poor asymptotic performance, and the median estimator may not be consistent. Oracle estimators with better asymptotic properties in this setting are the limited information maximum likelihood (LIML) estimator, see Bekker (1994) and Hansen, Hausman and Newey (2008), or the continuous updating estimator (CUE), see Newey and Windmeijer (2009). Selection of invalid instruments in this setting is outside the scope of this article.

## 3. Irrepresentable Condition

As $Z^{'}M_{\hat{d}}M_{\hat{d}}P_{Z}y=Z^{'}M_{\hat{d}}P_{Z}y=Z^{'}M_{\hat{d}}y$, it follows that

$$\begin{array}{ccc} ∥M_{\hat{d}}\left(P_{Z}y-Z\alpha \right){∥}_{2}^{2} & = & y^{'}P_{Z}M_{\hat{d}}P_{Z}y-2y^{'}M_{\hat{d}}Z\alpha +\alpha^{'}Z^{'}M_{\hat{d}}Z\alpha \\ & = & y^{'}P_{Z}M_{\hat{d}}P_{Z}y-2y^{'}\tilde{Z}{\alpha +\alpha }^{'}{\tilde{Z}}^{'}\tilde{Z}\alpha , \end{array}$$

where $\tilde{Z}=M_{\hat{d}}Z$. As

$$∥y-\tilde{Z}{\alpha ∥}_{2}^{2}=y^{'}y-2y^{'}\tilde{Z}{\alpha +\alpha }^{'}{\tilde{Z}}^{'}\tilde{Z}\alpha ,$$

it follows that the Lasso estimator ${\hat{\alpha }}^{\left(n\right)}$ as defined in (11) can equivalently be obtained as

(13) $${\hat{\alpha }}^{\left(n\right)}=\arg\min_{\alpha }\frac{1}{2}∥y-\tilde{Z}{\alpha ∥}_{2}^{2}+\lambda_{n}{∥\alpha ∥}_{1}.$$

This minimization problem looks very much like a standard Lasso approach with $\tilde{Z}$ as explanatory variables. However, an important difference is that $\tilde{Z}$ does not have full rank, but its rank is equal to *L* − 1. This is related to the standard Lasso case where we have an overcomplete dictionary implying that the OLS solution is not feasible. Intuitively, we cannot set λ_*n*_ = 0 in (13) as we have to shrink at least one element of ***α*** to zero to identify the parameter β. All just-identified models with *L* − 1 instruments included as invalid result in a residual correlation of 0, and hence setting λ_*n*_ = 0 does not lead to a unique 2SLS estimator.

We assume throughout that $E[{\tilde{Z}}_{i.}{\tilde{Z}}_{i.}^{'}]$ is finite. Let $C=\mathrm{plim}(n^{-1}{\tilde{Z}}^{'}\tilde{Z})$ , then it follows from Assumptions 1, 3, and 4 that **C** = ***Q − Qγ***(***γ***′***Qγ***)^− 1^***γ***′**Q** is finite.

We follow Zhao and Yu (2006) and Zou (2006), who developed the irrepresentable conditions for consistent Lasso variable selection. As before, let *A* = {*j*: α_*j*_ ≠ 0} and assume wlog that *A* = {1, 2, …, *s*}, *s* < *L*. (We will use subscripts *A* and 1 interchangeably from here onward, and subscript 2 for associations with the set *A^c^* = {*j*: α_*j*_ = 0}.) Let

(14) $$C=\left[\begin{array}{cc} C_{11} & C_{21}^{'}\\ C_{21} & C_{22} \end{array}\right],$$

where **C**_11_ is an *s* × *s* matrix. Further, define ${\hat{A}}_{n}=\left\{j:{\hat{\alpha }}_{j}^{\left(n\right)}\neq 0\right\}$. Let **s**(***α***_1_) denote the vector sgn(***α***_1_), where ***α***_1_ = ***α***_*A*_ = (α_1_, …, α_*s*_)′, sgn(*a*) = 1 if *a* > 0, sgn(*a*) = −1 if *a* < 0, and sgn(*a*) = 0 if *a* = 0. The irrepresentable condition

(15) $${∥C_{21}C_{11}^{-1}s\left(\alpha_{1}\right)∥}_{\infty }<1,$$

is an (almost) necessary and sufficient condition for consistent Lasso variable selection. While (15) refers to the formulation of the weak irrepresentable condition of Zhao and Yu (2006), they showed that in this setting of a random design with fixed *L* and constant parameters ***α***, their strong and weak irrepresentable conditions are equivalent to (15) almost surely (Zhao and Yu 2006, p. 2544).

If (15) is satisfied, and if λ_*n*_ satisfies λ_*n*_/*n* → 0 and $\lambda_{n}/\sqrt{n}\to \infty$, then $\lim_{n\to \infty }P\left({\hat{A}}_{n}=A\right)=1$, see Theorem 1 in Zhao and Yu (2006). Necessity means that consistent model selection implies the irrepresentable condition. As Zou (2006) showed, if $\lim_{n\to \infty }P\left({\hat{A}}_{n}=A\right)=1$ and under the same conditions λ_*n*_/*n* → 0 and $\lambda_{n}/\sqrt{n}\to \infty$, then the following condition must hold

(16) $${∥C_{21}C_{11}^{-1}s\left(\alpha_{1}\right)∥}_{\infty }\leq 1.$$

While in the standard linear model setup λ_*n*_/*n* → 0 guarantees estimation consistency, see Lemma 1 in Zou (2006), this is not the case in the IV setup here because of the rank deficiency of $\tilde{Z}$. Choosing λ_*n*_ = 0 in the standard setup would simply result in consistent OLS estimation of a model that includes all variables, which is not possible here as discussed above. Therefore, if the necessary irrepresentable condition (16) does not hold, consistent Lasso selection is not possible and even λ_*n*_/*n* → 0 does not guarantee estimation consistency in this rank deficient IV case.

We now analyze under what conditions the irrepresentable condition does or does not hold in the IV setup, focusing particularly on the relative strengths ***γ***_1_ and ***γ***_2_ of the invalid and valid instruments.

Partition **Q** = plim(*n*^− 1^**Z**′**Z**) and ***γ*** commensurate with the partitioning of **C** as

(17) $$Q=\left[\begin{array}{cc} Q_{11} & Q_{21}^{'}\\ Q_{21} & Q_{22} \end{array}\right],\;\gamma =\left(\begin{array}{c} \gamma_{1}\\ \gamma_{2} \end{array}\right),$$

where the instruments have been standardized such the diagonal elements of **Q** are equal to 1. In contrast to **C**, **Q** is not rank deficient. Then for the Lasso specification (13), we have the following result.

Proposition 1. Consider the observational models (3) and (8) under Assumptions 1, 3, and 4. Let $C=\mathrm{plim}(n^{-1}{\tilde{Z}}^{'}\tilde{Z})$; **Q** = plim(*n*^− 1^**Z**′**Z**); and **C**_11_, **C**_21_, **Q**_11_, **Q**_21_, **Q**_22_, ***γ***_1_, and ***γ***_2_ as specified in (14) and (17 ). Then **C**_21_**C**^− 1^_11_ is given by

(18) $$C_{21}C_{11}^{-1}=Q_{21}Q_{11}^{-1}-{\tilde{Q}}_{22}\gamma_{2}\frac{\gamma_{1}^{'}+\gamma_{2}^{'}Q_{21}Q_{11}^{-1}}{\gamma_{2}^{'}{\tilde{Q}}_{22}\gamma_{2}}\text{,}$$

where

Q˜22=Q22-Q21Q11-1Q21'= plim n-1Z2'MZ1Z2.

Proof. See Section A.1 in the supplementary materials.

Proposition 1 shows that consistent selection of the instruments is not only affected by the correlation structure of the instruments, but also by the values of ***γ***_1_ and ***γ***_2_. The next Proposition derives conditions on ***γ***_1_ and ***γ***_2_ under which the necessary condition for consistent variable selection (16) does not hold.

Proposition 2. Under the assumptions of Proposition 1, if |***γ***′_1_**s**(***α***_1_)| > ‖***γ***_2_‖_1_, then ‖**C**_21_**C**^− 1^_11_**s**(***α***_1_)‖_∞_ > 1.

Proof. It follows from (18) that

$$\left|\gamma_{2}^{'}C_{21}C_{11}^{-1}s\left(\alpha_{1}\right)\right|=\left|\gamma_{1}^{'}s\left(\alpha_{1}\right)\right|\text{.}$$

Therefore,

$$\begin{array}{ccc} {∥\gamma_{2}∥}_{1}{∥C_{21}C_{11}^{-1}s\left(\alpha_{1}\right)∥}_{\infty } & \geq & \left|\gamma_{1}^{'}s\left(\alpha_{1}\right)\right|\\ {∥C_{21}C_{11}^{-1}s\left(\alpha_{1}\right)∥}_{\infty } & \geq & \frac{\left|\gamma_{1}^{'}s\left(\alpha_{1}\right)\right|}{{∥\gamma_{2}∥}_{1}}\text{.} \end{array}$$

Hence, ‖**C**_21_**C**^− 1^_11_**s**(***α***_1_)‖_∞_ > 1 if |***γ***′_1_**s**(***α***_1_)| > ‖***γ***_2_‖_1_.

Remark 1. If **s**(***α***_1_) = **s**(***γ***_1_), then |***γ***′_1_**s**(***α***_1_)| = ‖***γ***_1_‖_1_, its maximum. Regardless of the correlation structure of the instruments, ‖**C**_21_**C**^− 1^_11_**s**(***α***_1_)‖_∞_ > 1 and hence the necessary condition for consistent Lasso variable selection does not hold in that case if ‖***γ***_1_‖_1_ > ‖***γ***_2_‖_1_, that is, when the invalid instruments are stronger (in *l*_1_-norm) than the valid ones.

From Proposition 1, we can investigate consistent selection for various cases of interest. Related to the Monte Carlo simulations in KZCS and in Section 5, Corollary 1 considers the case with $\gamma_{1}={\tilde{\gamma }}_{1}\iota_{s}$ and $\gamma_{2}={\tilde{\gamma }}_{2}\iota_{L-s}$.

Corollary 1. If $\gamma_{1}={\tilde{\gamma }}_{1}\iota_{s}$ and $\gamma_{2}={\tilde{\gamma }}_{2}\iota_{L-s}$, then |***γ***′_1_**s**(***α***_1_)| > ‖***γ***_2_‖_1_ if $|\frac{{\tilde{\gamma }}_{1}}{{\tilde{\gamma }}_{2}}\left|\right|\iota_{s}^{'}s\left(\alpha_{1}\right)|>L-s$. Let *g* = |***ι***′_*s*_**s**(***α***_1_)|, then it follows that ‖**C**_21_**C**^− 1^_11_**s**(***α***_1_)‖_∞_ > 1 if $|\frac{{\tilde{\gamma }}_{1}}{{\tilde{\gamma }}_{2}}|g>L-s$. Hence, if *g* = *s*, ||**C**_21_**C**^− 1^_11_**s**(***α***_1_)||_∞_ > 1 if $s>L/(1+|\frac{{\tilde{\gamma }}_{1}}{{\tilde{\gamma }}_{2}}|)$.

When instruments are uncorrelated, such that **Q = I**_*L*_**,** it follows that ‖**C**_21_**C**^− 1^_11_**s**(***α***_1_)‖_∞_ < 1 if $s<L-|\frac{{\tilde{\gamma }}_{1}}{{\tilde{\gamma }}_{2}}|g$. Hence, if *g* = *s*, ||**C**_21_**C**^− 1^_11_**s**(***α***_1_)||_∞_ < 1 if $s<L/(1+|\frac{{\tilde{\gamma }}_{1}}{{\tilde{\gamma }}_{2}}|)$.

Remark 2. For equal strength instruments, ${\tilde{\gamma }}_{1}={\tilde{\gamma }}_{2}$, the result of Corollary 1 shows that the necessary condition (16) does not hold for all possible configurations of ***α***_1_ if *s* > *L*/2. For uncorrelated equal strength instruments, the irrepresentable condition (15) holds for all possible configurations of ***α***_1_ if *s* < *L*/2.

## 4. A Consistent Estimator when *s* < *L*/2 and Adaptive Lasso

As the results above highlight, the Lasso path may not include the correct model, leading to an inconsistent estimator of β. This is the case even if less than 50% of the instruments are invalid because of differential instrument strength and/or correlation patterns of the instruments. Indeed, we find in the simulation exercise of Section 5.1 that the Lasso selects the valid instruments as invalid if these are relatively weak, ‖***γ***_2_‖_1_ < ‖***γ***_1_‖_1_, for a design with **s**(***α***_1_) = **s**(***γ***_1_). In this section, we present an estimation method that consistently selects the invalid instruments when less than 50% of the potential instruments are invalid. This is the same condition as that for the Lasso selection problem to satisfy the irrepresentable condition for equal strength uncorrelated instruments, but the proposed estimator below is consistent when the instruments have differential strength and/or have a general correlation structure.

We consider the adaptive Lasso approach of Zou (2006) using an initial consistent estimator of the parameters. In the standard linear case, the OLS estimator in the model with all explanatory variables included is consistent. As explained in Section 3, in the instrumental variables model this option is not available. We build on the result of Han (2008), who shows that the median of the *L* IV estimates of β using one instrument at the time is a consistent estimator of β in a model with invalid instruments, but where the instruments cannot have direct effects on the outcome, unless the instruments are uncorrelated.

Let $\hat{\Gamma }={\left(Z^{'}Z\right)}^{-1}Z^{'}y$; $\hat{\gamma }={\left(Z^{'}Z\right)}^{-1}Z^{'}d$ and let $\hat{\pi }$ be the *L*-vector with *j*th element

(19) $${\hat{\pi }}_{j}=\frac{{\hat{\Gamma }}_{j}}{{\hat{\gamma }}_{j}}.$$

Under the standard assumptions, Theorem 1 shows that the median of the ${\hat{\pi }}_{j}$, denoted ${\hat{\beta }}_{m}$, is a consistent estimator for β when *s* < *L*/2, without any further restrictions on the relative strengths or correlations of the instruments. Theorem 1 also shows that $\sqrt{n}\left({\hat{\beta }}_{m}-\beta \right)$ converges in distribution to that of an order statistic. From these results it follows that the consistent estimator ${\hat{\alpha }}_{m}=\hat{\Gamma }-\hat{\gamma }{\hat{\beta }}_{m}$ can be used for the adaptive Lasso approach of Zou (2006), resulting in oracle properties of the resulting estimator of β.

Theorem 1. Under model specifications (3) and (8) with Assumptions 1–4, let $\hat{\pi }$ be the *L*-vector with elements as defined in (19). If *s* < *L*/2, then the estimator ${\hat{\beta }}_{m}$ defined as

β^m= median π^

is a consistent estimator for β,

plim β^m=β.

Let ${\hat{\pi }}_{2}$ be the *L* − *s* vector with elements ${\hat{\pi }}_{j}$, *j* = *s* + 1, …, *L*. The limiting distribution of ${\hat{\beta }}_{m}$ is given by

$$\sqrt{n}\left({\hat{\beta }}_{m}-\beta \right)\overset{d}{⟶}q_{\left[l\right],L-s},$$

where for *L* odd, *q*_[*l*], *L* − *s*_ is the *l*th-order statistic of the limiting normal distribution of $\sqrt{n}\left({\hat{\pi }}_{2}-\beta \iota_{L-s}\right)$, where *l* is determined by *L*, *s*, and the signs of $\delta_{j}=\frac{\alpha_{j}}{\gamma_{j}}$, *j* = 1, …, *s*. For *L* even, *q*_[*l*], *L* − *s*_ is defined as the average of either the [*l*] and [*l* − 1]-order statistics, or the [*l*] and [*l* + 1]-order statistics.

Proof. See Section A.2 in the supplementary materials.

Given the consistent estimator ${\hat{\beta }}_{m}$, we obtain a consistent estimator for ***α*** as

$${\hat{\alpha }}_{m}={\left(Z^{'}Z\right)}^{-1}Z^{'}\left(y-d{\hat{\beta }}_{m}\right)=\hat{\Gamma }-\hat{\gamma }{\hat{\beta }}_{m},$$

which can then be used for the adaptive Lasso specification of (13) as proposed by Zou (2006). The adaptive Lasso estimator for ***α*** is defined as

(20) $${\hat{\alpha }}_{ad}^{\left(n\right)}=\arg\min_{\alpha }\frac{1}{2}{∥y-\tilde{Z}\alpha ∥}_{2}^{2}+\lambda_{n}\sum_{l=1}^{L}\frac{\left|\alpha_{l}\right|}{{\left|{\hat{\alpha }}_{m,l}\right|}^{\upsilon }},$$

and, for given values of υ can be estimated straightforwardly using the LARS algorithm, see Zou (2006). The resulting adaptive Lasso estimator for β is obtained as

$${\hat{\beta }}_{\text{ad}}^{\left(n\right)}=\frac{{\hat{d}}^{'}\left(y-Z{\hat{\alpha }}_{\text{ad}}^{\left(n\right)}\right)}{{\hat{d}}^{'}\hat{d}}.$$

As the result for the limiting distribution of the median estimator shows, ${\hat{\beta }}_{m}$, although converging at the $\sqrt{n}$ rate, has an asymptotic bias. This clearly also results in an asymptotic bias of ${\hat{\alpha }}_{m}$. As $\sqrt{n}\left({\hat{\alpha }}_{m}-\alpha \right)=O_{p}\left(1\right)$, Theorem 2 together with Remark 1 in Zou (2006) states the following properties of the adaptive Lasso estimator ${\hat{\alpha }}_{\text{ad}}^{\left(n\right)}$, where ${\hat{A}}_{\text{ad},n}=\left\{j:{\hat{\alpha }}_{\text{ad},j}^{\left(n\right)}\neq 0\right\}$.

Proposition 3. Suppose that $\lambda_{n}=o\left(\sqrt{n}\right)$ and ${\left(\sqrt{n}\right)}^{\nu -1}\lambda_{n}\to \infty$, then the adaptive Lasso estimator ${\hat{\alpha }}_{ad}^{\left(n\right)}$ satisfies

1. Consistency in variable selection: $\lim_{n\to \infty }P\left({\hat{A}}_{ad,n}=A\right)=1.$
2. Asymptotic normality: $\sqrt{n}\left({\hat{\alpha }}_{ad,A}^{\left(n\right)}-\alpha_{A}\right)\overset{d}{⟶}N\left(0,\sigma^{2}C_{11}^{-1}\right).$

Proof. See Zou (2006), Theorem 2 and Remark 1.

From the results of Proposition 3, it follows that the limiting distribution of ${\hat{\beta }}_{\text{ad}}^{\left(n\right)}$ is that of the oracle 2SLS estimator of β, as stated in the next Corollary.

Corollary 2. Under the conditions of Proposition 3, the limiting distribution of the adaptive Lasso estimator ${\hat{\beta }}_{\text{ad}}^{\left(n\right)}$ is given by

(21) $$\sqrt{n}({\hat{\beta }}_{\text{ad}}^{\left(n\right)}-\beta )\overset{d}{⟶}N(0,\sigma_{\beta_{\text{or}}}^{2}),$$

with $\sigma_{\beta_{\text{or}}}^{2}$ as defined in (7).

## 5. Simulation Results

### 5.1. Relative Strength of Instruments

We start with presenting some estimation results from a Monte Carlo exercise which is similar to that in KZCS. The data are generated from

$$\begin{array}{ccc} Y_{i} & = & D_{i}\beta +Z_{i.}^{'}\alpha +\varepsilon_{i}\\ D_{i} & = & Z_{i.}^{'}\gamma +v_{i}, \end{array}$$

where

$$\begin{array}{ccc} \left(\begin{array}{c} \varepsilon_{i}\\ v_{i} \end{array}\right) & ∼ & N\left(\left(\begin{array}{c} 0\\ 0 \end{array}\right),\left(\begin{array}{cc} 1 & \rho \\ \rho & 1 \end{array}\right)\right);\\ Z_{i.} & ∼ & N(0,I_{L}); \end{array}$$

and we set β = 0; *L* = 10; ρ = 0.25; *s* = 3, and the first *s* elements of ***α*** are equal to *a* = 0.2. Further, $\gamma_{1}={\tilde{\gamma }}_{1}\iota_{s}$ and $\gamma_{2}={\tilde{\gamma }}_{2}\iota_{L-s}$. Note that none of the estimation results presented here and below depend on the value of β. Table 1 presents estimation results for estimators of β in terms of bias, standard deviation, root mean squared error (rmse), and median absolute deviation (mad) for 1000 replications for sample sizes of *n* = 500, *n* = 2000, and *n* = 10, 000 for an equal strength design, with ${\tilde{\gamma }}_{1}={\tilde{\gamma }}_{2}=0.2$.

**Table 1.** Estimation results for 2SLS and Lasso estimators for β; *L* = 10, *s* = 3, ${\tilde{\gamma }}_{1}={\tilde{\gamma }}_{2}$.

					av. # instr	freq. all	
					selected as invalid	invalid instr	
β	bias	std dev	rmse	mad	[min, max]	selected	
*n* = 500							
2SLS	0.2966	0.0808	0.3074	0.2944	0	0	
2SLS or	0.0063	0.0843	0.0845	0.0570	3	1	
Lasso${}_{\text{cv}}$	0.1384	0.0965	0.1687	0.1352	6.41 [2,9]	0.990	
Post-Lasso${}_{\text{cv}}$	0.1169	0.1136	0.1630	0.1143			
Lasso${}_{\text{cvse}}$	0.2206	0.0847	0.2363	0.2174	3.16 [0,8]	0.664	
Post-Lasso${}_{\text{cvse}}$	0.0905	0.1243	0.1537	0.0994			
*n* = 2000							
2SLS	0.3019	0.0387	0.3044	0.3007	0	0	
2SLS or	0.0047	0.0422	0.0424	0.0285	3	1	
Lasso${}_{\text{cv}}$	0.0721	0.0509	0.0882	0.0705	6.64 [3,9]	1	
Post-Lasso${}_{\text{cv}}$	0.0617	0.0577	0.0845	0.0644			
Lasso${}_{\text{cvse}}$	0.1140	0.0430	0.1218	0.1165	3.76 [3,8]	1	
Post-Lasso${}_{\text{cvse}}$	0.0277	0.0521	0.0590	0.0387			
*n* = 10, 000							
2SLS	0.2996	0.0177	0.3002	0.2992	0	0	
2SLS or	0.0006	0.0182	0.0182	0.0126	3	1	
Lasso${}_{\text{cv}}$	0.0317	0.0236	0.0395	0.0311	6.44 [3,9]	1	
Post-Lasso${}_{\text{cv}}$	0.0272	0.0267	0.0380	0.0282			
Lasso${}_{\text{cvse}}$	0.0479	0.0187	0.0514	0.0489	3.81 [3,9]	1	
Post-Lasso${}_{\text{cvse}}$	0.0118	0.0238	0.0265	0.0176			

NOTE: Results from 1000 MC replications; β = 0; ρ = 0.25; *a* = 0.2; ${\tilde{\gamma }}_{2}=0.2$.

The information content for IV estimation can be summarized by the concentration parameter, see Rothenberg (1984). For the oracle estimation of β by 2SLS, the concentration parameter is given by μ^2^_*n*_ = ***γ***_2_′**Z**′_2_**M**_*Z*_1__**Z**_2_***γ***_2_/σ^2^_*v*_. For this data-generating process with independent instruments, the concentration parameter is therefore approximately *n*(*L* − *s*)(0.2^2^) and hence equal to 140 , 560, and 2800 for the three sample sizes. μ^2^_*n*_ can be seen as a population Wald statistic for testing *H*_0_: ***γ***_2_ = 0. The corresponding population *F*-statistics are equal to *n*(0.2^2^), or 20, 80, and 400 for the sample sizes 500, 2000, and 10,000, respectively.

A summary measure of the information content for Lasso selection is the (squared) signal-to-noise ratio (SNR), denoted by η^2^. It is defined as

$$\eta^{2}=\frac{\alpha_{1}^{'}C_{11}\alpha_{1}}{\sigma_{\varepsilon }^{2}}\text{,}$$

see, for example, Bühlmann and van der Geer (2011, p. 25). Analogously to the concentration parameter, *n*η^2^ can be interpreted as a population Wald statistic for testing *H*_0_: ***α***_1_ = 0. We analyze the effects of varying μ^2^_*n*_ and η^2^ more extensively in Section B.2 in the supplementary materials, where we derive that, for this design,

(22) $$\eta^{2}=\frac{\left(L-s\right)a^{2}}{{\left(\frac{{\tilde{\gamma }}_{1}}{{\tilde{\gamma }}_{2}}\right)}^{2}+\frac{L-s}{s}},$$

resulting in η^2^ = 0.084 for the parameter values considered in Table 1.

The “2SLS” results are for the naive 2SLS estimator of β that treats all instruments as valid. The probability limit of this estimator is given by

(23) plim β^naive=β+γ'Qαγ'Qγ=β+γ1'Q11α1+γ2'Q21α1γ1'Q11γ1+2γ2'Q21γ1+γ2'Q22γ2.

Therefore, in the design specified here, we have $\mathrm{plim}({\hat{\beta }}_{\text{naive}})=s/L=0.3.$

The “2SLS or” is the oracle 2SLS estimator that correctly includes the three invalid instruments in the model as explanatory variables. For the Lasso estimates, the value for λ_*n*_ has been obtained by 10-fold cross-validation, using the one-standard error rule, as in KZCS. This estimator is denoted “Lasso${}_{\text{cvse}}$” and is the one produced by the *sisVIVE* routine. We also present results for the cross-validated estimator that does not use the one-standard error rule, denoted “Lasso${}_{\text{cv.}}$” For the Lasso estimation procedure, we standardize throughout such that the diagonal elements of ${\tilde{Z}}^{'}\tilde{Z}/n$ are equal to 1.

We further present results for the so-called post-Lasso estimator, see, for example, Belloni et al. (2012), which is called the LARS-OLS hybrid by Efron et al. (2004). This is here simply the 2SLS estimator in the model that includes $Z_{{\hat{A}}_{n}}$ , the set of instruments with nonzero estimated Lasso coefficients. Clearly, when ${\hat{A}}_{n}=A$, the post-Lasso 2SLS estimator is equal to the oracle 2SLS estimator. The post-Lasso 2SLS estimator is expected to have a smaller bias as it avoids the bias in the Lasso estimate of β due to the shrinkage of the Lasso estimate of ***α*** toward **0**, see also Hastie, Tibshirani, and Friedman (2009, p. 91). This shrinkage bias effect on ${\hat{\beta }}^{\left(n\right)}$ for models where $A\subseteq {\hat{A}}_{n}$ is in the direction of the bias of ${\hat{\beta }}_{\text{naive}}$, where ***α*** is assumed to be **0**. (In an OLS setting, Belloni and Chernozhukov (2013) showed that the post-Lasso estimator can perform at least as well as Lasso in terms of rate of convergence, but is less biased even if the Lasso-based model selection misses some components of the true model.)

Further entries in Table 1 are the average number of instruments selected as invalid, that is, the average number of instruments in ${\hat{A}}_{n}=\left\{j:{\hat{\alpha }}_{j}^{\left(n\right)}\neq 0\right\}$, together with the minimum and maximum number of selected instruments, and the proportion of times the instruments selected as invalid include all three invalid instruments.

The results in Table 1 reveal some interesting patterns. First of all, the Lasso_*cv*_ estimator outperforms the Lasso${}_{\text{cvse}}$ estimator in terms of bias, rmse, and mad for all sample sizes, but this is reversed for the post-Lasso estimators, that is, the post-Lasso${}_{\text{cvse}}$ outperforms the post-Lasso${}_{\text{cv}}$. The Lasso${}_{\text{cv}}$ estimator selects on average around 6.5 instruments as invalid, which is virtually independent of the sample size. The Lasso${}_{\text{cvse}}$ estimator selects on average around 3.8 instruments as invalid for *n* = 2000 and *n* = 10, 000, but fewer, 3.16 for *n* = 500. Although the three invalid instruments are always jointly selected as invalid for the larger sample sizes, the Lasso${}_{\text{cvse}}$ is substantially biased, the biases being larger than twice the standard deviations. The post-Lasso${}_{\text{cvse}}$ estimator performs best, but is still outperformed by the oracle 2SLS estimator at *n* = 10, 000. Although the post-Lasso${}_{\text{cvse}}$ estimator has a larger standard deviation than the Lasso${}_{\text{cvse}}$ estimator, it has a smaller bias, rmse, and mad for all sample sizes.

We focus below on the performance of the median and adaptive Lasso estimators for a design with invalid instruments that are stronger than the valid ones, but for comparison we present results for these estimators for this equal strength instruments design in Section B.1 in the supplementary materials, which also includes a more detailed analysis of the differences in performances of the Lasso and post-Lasso estimators in this design.

Table 2 presents estimation results for the same Monte Carlo design as in Table 1, but now with stronger invalid than valid instruments, with ${\tilde{\gamma }}_{2}=0.2$ and ${\tilde{\gamma }}_{1}=3{\tilde{\gamma }}_{2}$. At these relative values, the necessary condition (16) is not satisfied and the Lasso selection will here select the valid instruments as invalid. Note that the behavior of the oracle 2SLS estimator is the same as in Table 1. In this case, $\beta +a/{\tilde{\gamma }}_{2}=0+0.2/0.6=0.33$ , which is the parameter value estimated by the invalid instruments. From (22), it follows that the SNR is smaller here, with η^2^ = 0.0247. The estimation results for the adaptive Lasso are based on setting υ=1. The resulting estimators are denoted as “ALasso.” As *L* is even here, the median is defined as ${\hat{\beta }}_{m}=\left({\hat{\pi }}_{\left[5\right]}+{\hat{\pi }}_{\left[6\right]}\right)/2$, where ${\hat{\pi }}_{\left[j\right]}$ is the *j*th-order statistic.

**Table 2.** Estimation results for estimators of β; *L* = 10, $s=3,{\tilde{\gamma }}_{1}=3{\tilde{\gamma }}_{2}$.

					av. # instr	freq. all
					selected as invalid	invalid instr
β	bias	std dev	rmse	mad	[min, max]	selected
*n* = 500						
Post-Lasso${}_{\text{cv}}$	0.2696	0.0583	0.2759	0.2718	5.06 [0,9]	0.03
Post-Lasso${}_{\text{cvse}}$	0.2658	0.0429	0.2692	0.2651	0.45 [0,8]	0
${\hat{\beta }}_{m}$	0.1128	0.0936	0.1466	0.1129		
ALasso${}_{\text{cv}}$	0.1735	0.0952	0.1979	0.1830	3.73 [0,9]	0.48
Post-ALasso${}_{\text{cv}}$	0.1324	0.1321	0.1870	0.1591		
ALasso${}_{\text{cvse}}$	0.2586	0.0420	0.2620	0.2568	0.46 [0,6]	0.04
Post-ALasso${}_{\text{cvse}}$	0.2428	0.0787	0.2552	0.2568		
*n* = 2000						
Post-Lasso${}_{\text{cv}}$	0.3004	0.0308	0.3020	0.3023	8.89 [3,9]	0.01
Post-Lasso${}_{\text{cvse}}$	0.2910	0.0352	0.2931	0.2932	6.58 [0,9]	0.00
${\hat{\beta }}_{m}$	0.0634	0.0500	0.0808	0.0649		
ALasso${}_{\text{cv}}$	0.0600	0.0527	0.0798	0.0596	4.42 [3,9]	0.998
Post-ALasso${}_{\text{cv}}$	0.0360	0.0626	0.0722	0.0442		
ALasso${}_{\text{cvse}}$	0.1656	0.0489	0.1726	0.1668	3.07 [0,6]	0.89
Post-ALasso${}_{\text{cvse}}$	0.0281	0.0774	0.0823	0.0348		
*n* = 10, 000						
Post-Lasso${}_{\text{cv}}$	0.3197	0.0120	0.3199	0.3202	8.97 [8,9]	0
Post-Lasso${}_{\text{cvse}}$	0.3202	0.0122	0.3204	0.3204	8.70 [7,9]	0
${\hat{\beta }}_{m}$	0.0278	0.0226	0.0358	0.0284		
ALasso${}_{\text{cv}}$	0.0153	0.0222	0.0270	0.0190	3.92 [3,9]	1
Post-ALasso${}_{\text{cv}}$	0.0092	0.0253	0.0269	0.0177		
ALasso${}_{\text{cvse}}$	0.0661	0.0212	0.0694	0.0668	3.02 [3,6]	1
Post-ALasso${}_{\text{cvse}}$	0.0010	0.0186	0.0187	0.0129		

NOTE: Results from 1000 MC replications; *a* = 0.2; β = 0; ${\tilde{\gamma }}_{2}=0.2,$ ρ = 0.25.

The results in Table 2 confirm that, for large sample sizes, the Lasso selects the valid instruments as invalid because of the relative strength of the invalid instruments. The post-ALasso${}_{\text{cvse}}$ estimator does not perform well for *n* = 500, but does for the sample sizes of *n* = 2000, and *n* = 10, 000, with results for the latter very similar to the oracle 2SLS results. The Post-ALasso${}_{\text{cv}}$ estimator performs better at *n* = 500, as it selects more instruments as invalid with a larger proportion correctly selecting all invalid instruments, although it is outperformed there by the simple median estimator ${\hat{\beta }}_{m}$.

### 5.2. Alternative Stopping Rule

The results for the Lasso estimator in Table 1 show that the 10-fold cross-validation method tends to select too many valid instruments as invalid over and above the invalid ones, and that the ad hoc one-standard error rule does improve the selection. The fact that the cross-validation method selects too many variables is well known, see, for example, Bühlmann and van der Geer (2011), who argued that use of the cross-validation method is appropriate for prediction purposes, but that the penalty parameter needs to be larger for variable selection, as achieved by the one-standard error rule. Selecting valid instruments as invalid in addition to correctly selecting the invalid instruments clearly does not lead to an asymptotic bias, but results in a less efficient estimator as compared to the oracle estimator.

We propose a stopping rule for the LARS/Lasso algorithm based on the approach of Andrews (1999) for moment selection, which is particularly well-suited for the IV selection problem. We can use this approach because the number of instruments *L* ≪ *n*. This stopping rule is computationally less expensive than cross-validation.

Consider again the oracle model

(24) $$y=d\beta +Z_{A}\alpha_{A}+\varepsilon =R_{A}\theta_{A}+\varepsilon .$$

Let **g**_*n*_(***θ***_*A*_) = *n*^− 1^**Z**′(**y − R**_*A*_***θ***_*A*_), and **W**_*n*_ a *k_z_* × *k_z_* weight matrix, then the oracle generalized method of moments (GMM) estimator is defined as

$${\hat{\theta }}_{A,\text{gmm}}=\arg\min_{\theta_{A}}g_{n}{\left(\theta_{A}\right)}^{'}W_{n}^{-1}g_{n}\left(\theta_{A}\right),$$

see Hansen (1982). 2SLS is a one-step GMM estimator, setting **W**_*n*_ = *n*^− 1^**Z**′**Z**. Given the moment conditions *E*[**Z**_*i*._ϵ_*i*_] = 0, 2SLS is efficient under conditional homoscedasticity, *E*(ϵ^2^_*i*_|**Z**_*i*._) = σ^2^_ϵ_. Under general forms of conditional heteroscedasticity, an efficient two-step oracle GMM estimator is obtained by setting

$$W_{n}=W_{n}\left({\hat{\theta }}_{A,1}\right)=n^{-1}\sum_{i=1}^{n}({\left(y_{i}-R_{A,i.}^{'}{\hat{\theta }}_{A,1}\right)}^{2}Z_{i.}Z_{i.}^{'}),$$

where ${\hat{\theta }}_{A,1}$ is an initial consistent estimator, with a natural choice the 2SLS estimator. Then, under the null that the moment conditions are correct, *E*[**Z**_*i*._ϵ_*i*_] = 0, the Hansen (1982) *J*-test statistic and its limiting distribution are given by

$$\begin{array}{ccc} J_{n}\left({\hat{\theta }}_{A,\text{gmm}}\right) & = & ng_{n}{\left({\hat{\theta }}_{A,\text{gmm}}\right)}^{'}W_{n}^{-1}\left({\hat{\theta }}_{A,1}\right)g_{n}\left({\hat{\theta }}_{A,\text{gmm}}\right)\\ & \overset{d}{\to } & \chi_{\left(L-\dim\left(R_{A}\right)\right)}^{2}. \end{array}$$

For any set *A*^+^, such that *A*⊂*A*^+^, we have that

$$J_{n}\left({\hat{\theta }}_{A^{+},\text{gmm}}\right)\overset{d}{\to }\chi_{\left(L-\dim\left(R_{A^{+}}\right)\right)}^{2},$$

whereas for any set *A*^−^, such that $A\neg \subset A^{-}$, $J_{n}\left({\hat{\theta }}_{A^{-},\text{gmm}}\right)=O_{p}\left(n\right)$.

Note that the *J*-test is a robust score, or Lagrange multiplier, test for testing *H*_0_: ***α***_*C*_ = 0 in the just identified specification

$$y=d\beta +Z_{B}\alpha_{B}+Z_{C}\alpha_{C}+\varepsilon ,$$

where **Z**_*B*_ is a *k_B_* set of instruments included in the model and **Z**_*C*_ is any selection of *L* − *k_B_* − 1 instruments from the *L* − *k_B_* set of instruments not in **Z**_*B*_, see, for example, Davidson and MacKinnon (1993, p. 235). This makes clear the link between the *J*-test and testing for additional invalid instruments of the form as specified in model (3).

We can now combine the LARS/Lasso algorithm with the Hansen *J*-test, which is a directed downward testing procedure in the terminology of Andrews (1999). Compute $J_{n}\left({\hat{\theta }}_{{\hat{A}}_{n}^{\left[j\right]}}\right)$ at every LARS/Lasso step *j* = 0, 1, 2, …, where ${\hat{A}}_{n}^{\left[0\right]}=⌀$ and ${∥{\hat{A}}_{n}^{\left[1\right]}∥}_{0}=1$, compare it to a corresponding critical value ζ_*n*, *L* − *k*_ of the χ^2^_(*L* − *k*)_ distribution, where $k=\dim(R_{{\hat{A}}_{n}^{\left[j\right]}})$ . We then select the model with the largest degrees of freedom *L* − *k*, for which $J_{n}\left({\hat{\theta }}_{{\hat{A}}_{n}^{\left[j\right]}}\right)$ is smaller than the critical value. If two models of the same dimension pass the test, which can happen with a Lasso step, the model with the smallest value of the *J*-test gets selected. (If there is no empirical evidence at all for any invalid instruments, that is, if $J_{n}\left({\hat{\theta }}_{{\hat{A}}_{n}^{\left[0\right]}}\right)$ is smaller than its corresponding critical value, then the model with all instruments as valid gets selected.) Clearly, this approach is a post-Lasso approach, where the LARS/Lasso algorithm is used purely for selection of the invalid instruments. For consistent model selection, the critical values ζ_*n*, *L* − *k*_ need to satisfy

(25) $$\zeta_{n,L-k}\to \infty \;\text{for}\;n\to \infty \text{,}\;\text{and}\;\;\zeta_{n,L-k}=o\left(n\right),$$

see Andrews (1999).

As the oracle model is on the adaptive LARS/Lasso path in large samples, this approach leads to consistent selection, $\lim_{n\to \infty }P\left({\hat{A}}_{n,ah}^{ad}=A\right)=1$, the subscript ah standing for Andrews/Hansen. As Guo et al. (2018, Theorem 2) showed, consistent selection implies that the limiting distribution of the 2SLS estimator ${\hat{\beta }}_{{\hat{A}}_{n,\text{ah}}^{\text{ad}}}$ is the same as that of the oracle 2SLS estimator, that is, $\sqrt{n}\left({\hat{\beta }}_{{\hat{A}}_{n,\text{ah}}^{\text{ad}}}-\beta \right)\overset{d}{\to }N\left(0,\sigma_{\beta_{\text{or}}}^{2}\right)$. We call ${\hat{\beta }}_{{\hat{A}}_{n,\text{ah}}^{\text{ad}}}$ the post-ALasso${}_{\text{ah}}$ estimator. This approach also leads to consistent selection along the Lasso path when the irrepresentable condition (15) holds, resulting in oracle properties of the resulting post-Lasso${}_{\text{ah}}$ estimator.

Let ζ_*n*, *L* − *k*_ = χ^2^_*L* − *k*_(*p_n_*) be the 1 − *p_n_* quantile of the χ^2^_*L* − *k*_ distribution. Here, *p_n_* is the *p*-value of the test. This combination of the Andrews/Hansen method with the LARS/Lasso steps therefore results in having to choose a *p*-value *p_n_* instead of a penalty parameter λ_*n*_. Keeping *n* fixed, choosing a large value for *p_n_* leads to selecting a larger set as invalid instruments as compared to choosing a smaller value for *p_n_*. Finite sample inference will not be straightforward, as this method is essentially a sequential approach where the model at step *j* is only considered when the model at step *j* − 1 is rejected. Using the consistent selection properties, we will investigate the behavior of the Wald test in the next section and find in our simulation designs that this method performs quite well and similar to the ALasso${}_{\text{cvse}}$ method in the unequal instrument strength design, and also performs well using the post-Lasso${}_{\text{ah}}$ estimator for the equal strength design.

Table 3 presents the estimation results using this stopping rule as a selection device for the Lasso estimator for the design with equal strength instruments and the adaptive Lasso estimator for the unequal instrument strength design, as in Tables 1 and 2. We denote the resulting 2SLS estimators as ”post-(A)Lasso${}_{\text{ah}}$.” The *p*-values here are chosen as *p_n_* = 0.1/ln (*n*), following Belloni et al. (2012), and are equal to 0.0161, 0.0132, and 0.0109 for *n* equal to 500, 2000, and 10, 000, respectively. For the equal strength design, the ah approach selects too few invalid instruments for *n* = 500, resulting in an upward bias, with bias, std dev, rmse, and mad very similar to those of the post-Lasso${}_{\text{cvse}}$ estimator in Table 1. For *n* = 2000 and *n* = 10, 000, this post-Lasso procedure performs well with properties very similar to that of the oracle 2SLS estimator, and with smaller bias, rmse, and mad than the post-Lasso${}_{\text{cvse}}$ method. For the unequal strength design, for *n* = 10, 000 the results are virtually identical to those of the oracle and post-ALasso${}_{\text{cvse}}$ estimators, whereas the post-ALasso${}_{\text{ah}}$ estimator performs better in terms of bias, std dev, rmse, and mad than the post-ALasso${}_{\text{cvse}}$ estimator when *n* = 2000. Again, when *n* = 500, the method does not select the invalid instruments.

**Table 3.** Results for post-(A)Lasso${}_{\text{ah}}$ 2SLS estimators for β; *L* = 10, *s* = 3.

						av. # instr	freq. all	
						selected as invalid	invalid instr	
	*n*	bias	std dev	rmse	mad	[min, max]	selected	
post-Lasso${}_{\text{ah}}$								
${\tilde{\gamma }}_{1}={\tilde{\gamma }}_{2}$	500	0.0896	0.1252	0.1539	0.1007	2.56 [0,5]	0.391	
	2000	0.0055	0.0430	0.0434	0.0286	3.02 [3,5]	1	
	10,000	0.0009	0.0186	0.0186	0.0129	3.02 [3,5]	1	
post-ALasso${}_{\text{ah}}$								
${\tilde{\gamma }}_{1}=3{\tilde{\gamma }}_{2}$	500	0.2172	0.1091	0.2431	0.2471	0.86 [0,5]	0.07	
	2000	0.0173	0.0677	0.0699	0.0303	3.05 [1,5]	0.93	
	10,000	0.0008	0.0186	0.0186	0.0129	3.01 [3,5]	1	

NOTE: Results from 1000 MC replications; β = 0; *a* = 0.2; ${\tilde{\gamma }}_{2}=0.2$; ρ = 0.25.

### 5.3. Inference

From the limiting distribution result (21), a simple approach to estimating the asymptotic variance of the post-ALasso 2SLS estimator for β is by calculating the standard 2SLS variance estimator. The post-ALasso 2SLS estimator is given by

$${\hat{\beta }}_{\text{ad,post}}^{\left(n\right)}={\left({\hat{d}}^{'}M_{Z_{{\hat{A}}_{\text{ad},n}}}\hat{d}\right)}^{-1}{\hat{d}}^{'}M_{Z_{{\hat{A}}_{\text{ad},n}}}y$$

and its estimated variance given by

(26) $$\text{v}\hat{\text{a}}\text{r}\left({\hat{\beta }}_{\text{ad,post}}^{\left(n\right)}\right)={\hat{\sigma }}_{\varepsilon }^{2}{\left({\hat{d}}^{'}M_{Z_{{\hat{A}}_{\text{ad},n}}}\hat{d}\right)}^{-1},$$

where ${\hat{\sigma }}_{\varepsilon }^{2}={\hat{\varepsilon }}^{'}\hat{\varepsilon }/n$, $\hat{\varepsilon }=y-d{\hat{\beta }}_{\text{ad,post}}^{\left(n\right)}-Z_{{\hat{A}}_{\text{ad},n}}{\hat{\alpha }}_{{\hat{A}}_{\text{ad},n},\text{post}}^{\left(n\right)}$. Under the conditions of Proposition 3, the standard assumptions and conditional homoscedasticity, $n\text{v}\hat{\text{a}}\text{r}\left({\hat{\beta }}_{\text{ad,post}}^{\left(n\right)}\right)\overset{p}{\to }\sigma_{\beta_{\text{or}}}^{2}$. A standard robust version, robust to general forms of heteroscedasticity, is given by

$$\begin{array}{ccc} & & \text{v}\hat{\text{a}}{\text{r}}_{r}\left({\hat{\beta }}_{\text{ad,post}}^{\left(n\right)}\right)\\ & & \;={\left({\hat{d}}^{'}M_{Z_{{\hat{A}}_{\text{ad},n}}}\hat{d}\right)}^{-1}{\hat{d}}^{'}M_{Z_{{\hat{A}}_{\text{ad},n}}}\hat{H}M_{Z_{{\hat{A}}_{\text{ad},n}}}\hat{d}{\left({\hat{d}}^{'}M_{Z_{{\hat{A}}_{\text{ad},n}}}\hat{d}\right)}^{-1}, \end{array}$$

where $\hat{H}$ is an *n* × *n* diagonal matrix with diagonal elements ${\hat{H}}_{ii}={\hat{\varepsilon }}_{i}^{2}$, for *i* = 1, …, *n*. The robust Wald test for the null *H*_0_: β = β_0_ is then given by

$$W_{\beta ,r}=\frac{({\hat{\beta }}_{\text{ad,post}}^{\left(n\right)}-\beta_{0}{)}^{2}}{\text{v}\hat{\text{a}}{\text{r}}_{r}({\hat{\beta }}_{\text{ad,post}}^{\left(n\right)})}.$$

From the results for the post-ALasso${}_{\text{cvse}}$ and post-ALasso${}_{\text{ah}}$ estimators for the unequal strength instruments design as presented in Tables 2 and 3, respectively, one would expect this approach to work well for the large sample case, *n* = 10,000, as there the estimation results are very close to those of the oracle 2SLS estimator. The robust Wald test for the null *H*_0_: β = 0, the true value of β, at the 10% level for *n* = 10,000 has a rejection frequency of 9.3% and 9.2% for the post-ALasso${}_{\text{cvse}}$ and post-ALasso${}_{\text{ah}}$ estimators, respectively, very close to that of the robust Wald test based on the oracle 2SLS estimator, which has a rejection frequency of 9.0%.

**Figure 1.**
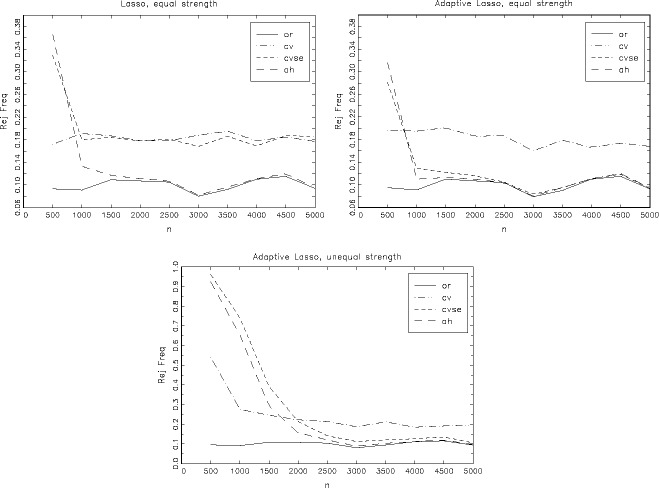
(a–c) Rejection frequencies of robust Wald tests for *H*_0_: β = 0 at 10% level as a function of sample size, in steps of 500. Equal strength instruments design, Post-Lasso in (a), Post-ALasso in (b). Unequal strength instruments design, Post-ALasso in (c). Based on 1000 MC replications for each sample size.

For the equal strength instruments design, we perform the same analysis for the post-Lasso estimators. Figures 1(a)–1(c) shows the performance of the robust Wald test *W*_β, *r*_, its rejection frequency at the 10% level, as a function of the sample size in steps of 500, *n* = 500, 1000, …, 5000. Figures 1(a) and 1(b) shows the results for the post-Lasso and post-ALasso estimators for the equal strength instruments design. Figure 1(c) shows the results for the post-ALasso estimators for the unequal strength instruments design.

Figure 1(a) clearly shows that the Lasso${}_{\text{cv}}$ and Lasso${}_{\text{cvse}}$ procedures do not result in consistent selection and the resulting post-Lasso estimators do not have oracle properties. The Wald test rejection frequencies remain constant for increasing sample size and larger than those of the oracle estimator. In contrast, the post-Lasso${}_{\text{ah}}$ estimator behaves very similar to the oracle estimator in this design from *n* = 1500 onward. Figure 1(b) shows that both the post-ALasso${}_{\text{cvse}}$ and post-ALasso${}_{\text{ah}}$ behave like the oracle estimator, again from *n* = 1500 onward in this design. The results in Figure 1(c) show that for the unequal instruments strength design considered here, the performances of the post-adaptive Lasso estimators are far from that of the oracle estimator in small samples, as expected from the results in Tables 2 and 3. The post-ALasso${}_{\text{ah}}$ behaves like the oracle estimator here from *n* = 4000 onward, with the post-ALasso${}_{\text{cvse}}$ estimator behaving similarly, but having a larger rejection frequency for all sample sizes considered here that are less than *n* = 5000.

The results in Tables 1–3 and Figures 1(a)–1(c) show clearly that the information content in the data, given the parameter values chosen here, is insufficient at *n* = 500 for the (adaptive) Lasso procedures to correctly select the invalid instruments and hence the resulting estimators have poor properties, far removed from those of the oracle estimator. At these levels of information, the ALasso${}_{\text{cv}}$ estimator is actually the preferred estimator as it counteracts the selection of too few invalid instruments of the ALasso${}_{\text{cvse}}$ and ALasso${}_{\text{ah}}$ estimators. We further explore how the performances of the estimators depend on the information content of the data-generating process in Section B.2 in the supplementary materials.

## 6. The Effect of BMI on Diastolic Blood Pressure Using Genetic Markers as Instruments

We use data on 105,276 individuals from the UK Biobank and investigate the effect of BMI on diastolic blood pressure (DBP). See Sudlow et al. (2015) for further information on the UK Biobank. We use 96 single nucleotide polymorphisms (SNPs) as instruments for BMI as identified in independent GWAS studies, see Locke et al. (2015).

With Mendelian randomization studies, the SNPs used as potential instruments can be invalid for various reasons, such as linkage disequilibrium, population stratification, and horizontal pleiotropy, see, for example, von Hinke et al. (2016) or Davey Smith and Hemani (2014). For example, an SNP has pleiotropic effects if it not only affects the exposure but also has a direct effect on the outcome. While we guard against population stratification by considering only white European origin individuals in our data, the use of the Lasso methods can be extremely useful here to identify the SNPs with direct effects on the outcome and to estimate the causal effect of BMI on diastolic blood pressure taking account of this.

Because of skewness, we log-transformed both BMI and DBP. The linear model specification includes age, age^2^, and sex, together with 15 principal components of the genetic relatedness matrix as additional explanatory variables. Table 4 presents the estimation results for the causal effect parameter, which is here the percentage change in DBP due to a 1% change in BMI. As *p*-value for the Hansen test-based procedures we take again 0.1/ln (*n*) = 0.0086.

**Table 4.** Estimation results, the effect of ln(BMI) on ln(DBP).

	estimate	rob st err	# instr	*p*-value *J*-test
			selected as invalid	
OLS	0.206	0.003		
2SLS	0.087	0.016	0	0.0000
Lasso${}_{\text{cv}}$	0.126		56	
Post-Lasso${}_{\text{cv}}$	0.145	0.033		1.0000
Lasso${}_{\text{cvse}}$	0.111		20	
Post-Lasso${}_{\text{cvse}}$	0.142	0.020		0.6435
Post-Lasso${}_{\text{ah}}$	0.122	0.018	12	0.0123
median, ${\hat{\beta }}_{m}$	0.148			
ALasso${}_{\text{cv}}$	0.158		54	
Post-ALasso${}_{\text{cv}}$	0.161	0.029		1.0000
ALasso${}_{\text{cvse}}$	0.131		17	
Post-ALasso${}_{\text{cvse}}$	0.151	0.019		0.4091
Post-ALasso${}_{\text{ah}}$	0.163	0.018	11	0.0102

NOTE: Sample size *n* = 105,276; *L* = 96.

The OLS estimate of the causal parameter is equal to 0.206 (s.e. 0.003), whereas the 2SLS estimate treating all 96 instruments as valid is much smaller at 0.087 (s.e. 0.016), with a 95% confidence interval of [0.056, 0.118]. The *J*-test, however, rejects the null that all the instruments are valid. The Lasso${}_{\text{cv}}$ estimator identifies a large number of 56 instruments as invalid and the Lasso${}_{\text{cv}}$ estimate is equal to 0.126, the post-Lasso${}_{\text{cv}}$ estimate is equal to 0.145. The Lasso${}_{\text{cvse}}$ procedure identifies 20 instruments as invalid and the Lasso${}_{\text{cvse}}$ estimate is equal to 0.111. The post-Lasso${}_{\text{cvse}}$ estimate is larger and equal to 0.142, which is in line with our findings above that the Lasso estimator is biased toward the 2SLS estimator that treats all instruments as valid due to shrinkage. The post-Lasso${}_{\text{ah}}$ procedure selects a subset of 12 instruments as invalid, and the post-Lasso${}_{\text{ah}}$ parameter estimate is equal to 0.122.

The median estimate ${\hat{\beta }}_{m}$ is equal to 0.148. Using this estimate for the adaptive Lasso results in the cv method selecting 54 instruments as invalid and the cvse method selecting 17 instruments as invalid. The adaptive Lasso${}_{\text{ah}}$ method selects a subset of 11 instruments as invalid. The post-ALasso${}_{\text{cv}}$, post-ALasso${}_{\text{cvse}}$, and post-ALasso${}_{\text{ah}}$ estimates are equal to 0.161, 0.151, and 0.163, respectively, with the 95% confidence intervals of the post-ALasso${}_{\text{cvse}}$ and post-ALasso${}_{\text{ah}}$ estimators given by [0.113,0.189] and [0.127,0.198 ], respectively. These results indicate that the OLS estimator is less confounded than suggested by the 2SLS estimation results using all 96 instruments as valid instruments.

The strongest potential instrument is the FTO SNP. For all Lasso estimators in Table 4, it is selected as an invalid instrument. The value for ${\hat{\pi }}_{\text{FTO}}=-0.009$, that is, negative, which is contrary to the direction of the found causal effect.

The *F*-test statistic for *H*_0_: ***γ***_2_ = 0 for the model resulting from the ALasso${}_{\text{ah}}$ procedure is equal to 18.21 with the associated estimate of the concentration parameter equal to 1547.81. The *F*-test result indicates that the 2SLS estimator may have some many weak instruments bias, see Stock and Yogo (2005). However, the LIML (limited information maximum likelihood) estimator in this model is very similar to the 2SLS estimator and is equal to 0.159 (s.e. 0.019), indicating that there is not a many weak instruments problem here, see Davies et al. (2015).

## 7. Conclusions

Instrumental variables estimation is a well-established procedure for the identification and estimation of causal effects of exposures on outcomes where the observed relationships are confounded by nonrandom selection of exposure. The main identifying assumption is that the instruments satisfy the exclusion restriction, that is, they only affect the outcomes through their relationship with the exposure. In an important contribution, Kang et al. (2016) showed that the Lasso method for variable selection can be used to select invalid instruments in linear IV models, even though there is no prior knowledge about which instruments are valid.

We have shown here that, even under the sufficient condition for identification that less than 50% of the instruments are invalid, the Lasso selection may select the valid instruments as invalid if the invalid instruments are relatively strong, that is, the case where an invalid instrument explains more of the exposure variance than a valid instrument. Consistent selection of invalid instruments also depends on the correlation structure of the instruments.

We show that a median estimator is consistent when less than 50% of the instruments are invalid, and its consistency does not depend on the relative strength of the instruments or their correlation structure. This initial consistent estimator can be used for the adaptive Lasso estimator of Zou (2006) and we show that it performs well for larger sample sizes/information settings in our simulations. This adaptive Lasso estimator has the same limiting distribution as the oracle 2SLS estimator, and solves the inconsistency problem of the Lasso method when the relative strength of the invalid instruments is such that the Lasso method selects the valid instruments as invalid.

## Supplementary Material

Supplemental Material

## Supplementary Materials

The document contains the proofs of Proposition 1 and Theorem 1 in Section A, and further simulation results and discussions in Section B.

The Stata module “SIVREG” implements the post-ALasso${}_{\text{ah}}$ method. Further details and documentation are provided in Farbmacher (2017).
