# Supplementary material for: On the Use of the Lasso for Instrumental Variables Estimation with Some Invalid Instruments
Source: J Am Stat Assoc. 2018 Nov 13;114(527):1339–50. doi: 10.1080/01621459.2018.1498346 (PMC6817329; doi:10.1080/01621459.2018.1498346)
Supplement: Supplemental Material [file UASA_A_1498346_SM7564.zip › UASA_A_1498346_Supplement/UASA_A_1498346_Supplement.pdf]

Supplementary Materials for "On the Use of the Lasso for Instrumental Variables Estimation with Some Invalid Instruments"

Frank Windmeijer[a,d,*], Helmut Farbmacher[b], Neil Davies[c,d]
George Davey Smith[c,d]

[a]Department of Economics, University of Bristol, UK

[b]Center for the Economics of Aging, Max Planck Society Munich, Germany

[c]School of Social and Community Medicine, University of Bristol, UK

[d]MRC Integrative Epidemiology Unit, Bristol, UK

April 2018

A Proofs

A.1 Proof of Proposition 1

Lemma 1 *For nonsingular $r \times r$ matrix $\mathbf{H}$ and r-vector $\mathbf{h}$, if $\mathbf{h}'\mathbf{H}^{-1}\mathbf{h} \neq 1$, then*

$$\left(\mathbf{H} - \mathbf{h}\mathbf{h}'\right)^{-1} = \mathbf{H}^{-1} + \frac{1}{1 - \mathbf{h}'\mathbf{H}^{-1}\mathbf{h}}\mathbf{H}^{-1}\mathbf{h}\mathbf{h}'\mathbf{H}^{-1} \tag{A.1}$$

and so

$$\begin{aligned} \mathbf{h}'\left(\mathbf{H} - \mathbf{h}\mathbf{h}'\right)^{-1} &= \left(1 + \frac{\mathbf{h}'\mathbf{H}^{-1}\mathbf{h}}{1 - \mathbf{h}'\mathbf{H}^{-1}\mathbf{h}}\right)\mathbf{h}'\mathbf{H}^{-1} \\ &= \frac{1}{1 - \mathbf{h}'\mathbf{H}^{-1}\mathbf{h}}\mathbf{h}'\mathbf{H}^{-1}. \end{aligned} \tag{A.2}$$

Proof. See Abadir and Magnus (2005, page 87). ■

From the definitions in Section 3, we have

$$\begin{aligned} \operatorname{plim}\left(\frac{1}{n}\widetilde{\mathbf{Z}}_1'\widetilde{\mathbf{Z}}_1\right) &= \mathbf{Q}_{11} - \left(\mathbf{Q}_{11}\boldsymbol{\gamma}_1 + \mathbf{Q}_{21}'\boldsymbol{\gamma}_2\right)\left(\boldsymbol{\gamma}'\mathbf{Q}\boldsymbol{\gamma}\right)^{-1}\left(\boldsymbol{\gamma}_1'\mathbf{Q}_{11} + \boldsymbol{\gamma}_2'\mathbf{Q}_{21}\right) \\ \operatorname{plim}\left(\frac{1}{n}\widetilde{\mathbf{Z}}_2'\widetilde{\mathbf{Z}}_1\right) &= \mathbf{Q}_{21} - \left(\mathbf{Q}_{21}\boldsymbol{\gamma}_1 + \mathbf{Q}_{22}\boldsymbol{\gamma}_2\right)\left(\boldsymbol{\gamma}'\mathbf{Q}\boldsymbol{\gamma}\right)^{-1}\left(\boldsymbol{\gamma}_1'\mathbf{Q}_{11} + \boldsymbol{\gamma}_2'\mathbf{Q}_{21}\right). \end{aligned}$$

*f.windmeijer@bristol.ac.uk

Hence

$$\begin{aligned}
\mathbf{C}_{21}\mathbf{C}_{11}^{-1} &= \operatorname{plim}\left(\frac{1}{n}\widetilde{\mathbf{Z}}_2'\widetilde{\mathbf{Z}}_1\right)\left(\operatorname{plim}\left(\frac{1}{n}\widetilde{\mathbf{Z}}_1'\widetilde{\mathbf{Z}}_1\right)\right)^{-1} \\
&= \left(\left(\boldsymbol{\gamma}'\mathbf{Q}\boldsymbol{\gamma}\right)\mathbf{Q}_{21} - \left(\mathbf{Q}_{21}\boldsymbol{\gamma}_1 + \mathbf{Q}_{22}\boldsymbol{\gamma}_2\right)\left(\boldsymbol{\gamma}_1'\mathbf{Q}_{11} + \boldsymbol{\gamma}_2'\mathbf{Q}_{21}\right)\right) \qquad \text{(A.3)} \\
&\quad \left(\left(\boldsymbol{\gamma}'\mathbf{Q}\boldsymbol{\gamma}\right)\mathbf{Q}_{11} - \left(\mathbf{Q}_{11}\boldsymbol{\gamma}_1 + \mathbf{Q}_{21}'\boldsymbol{\gamma}_2\right)\left(\boldsymbol{\gamma}_1'\mathbf{Q}_{11} + \boldsymbol{\gamma}_2'\mathbf{Q}_{21}\right)\right)^{-1}.
\end{aligned}$$

Let $\boldsymbol{\eta} = \left(\mathbf{Q}_{11}\boldsymbol{\gamma}_1 + \mathbf{Q}_{21}'\boldsymbol{\gamma}_2\right)$. From (A.2), we get

$$\begin{aligned}
\boldsymbol{\eta}'\left(\left(\boldsymbol{\gamma}'\mathbf{Q}\boldsymbol{\gamma}\right)\mathbf{Q}_{11} - \boldsymbol{\eta}\boldsymbol{\eta}'\right)^{-1} &= \frac{1}{1-\boldsymbol{\eta}'\mathbf{Q}_{11}^{-1}\boldsymbol{\eta}/\boldsymbol{\gamma}'\mathbf{Q}\boldsymbol{\gamma}}\boldsymbol{\eta}'\mathbf{Q}_{11}^{-1}/\boldsymbol{\gamma}'\mathbf{Q}\boldsymbol{\gamma} \\
&= \frac{1}{\boldsymbol{\gamma}'\mathbf{Q}\boldsymbol{\gamma} - \boldsymbol{\eta}'\mathbf{Q}_{11}^{-1}\boldsymbol{\eta}}\boldsymbol{\eta}'\mathbf{Q}_{11}^{-1}. \qquad \text{(A.4)}
\end{aligned}$$

Using (A.1),

$$\begin{aligned}
&\left(\boldsymbol{\gamma}'\mathbf{Q}\boldsymbol{\gamma}\right)\mathbf{Q}_{21}\left(\left(\boldsymbol{\gamma}'\mathbf{Q}\boldsymbol{\gamma}\right)\mathbf{Q}_{11} - \boldsymbol{\eta}\boldsymbol{\eta}'\right)^{-1} \\
= \;& \mathbf{Q}_{21}\mathbf{Q}_{11}^{-1} + \left(\boldsymbol{\gamma}'\mathbf{Q}\boldsymbol{\gamma}\right)\mathbf{Q}_{21}\frac{1}{1-\boldsymbol{\eta}'\mathbf{Q}_{11}^{-1}\boldsymbol{\eta}/\boldsymbol{\gamma}'\mathbf{Q}\boldsymbol{\gamma}}\mathbf{Q}_{11}^{-1}\boldsymbol{\eta}\boldsymbol{\eta}'\mathbf{Q}_{11}^{-1}/\left(\boldsymbol{\gamma}'\mathbf{Q}\boldsymbol{\gamma}\right)^2 \\
= \;& \mathbf{Q}_{21}\mathbf{Q}_{11}^{-1} + \mathbf{Q}_{21}\mathbf{Q}_{11}^{-1}\boldsymbol{\eta}\frac{1}{\boldsymbol{\gamma}'\mathbf{Q}\boldsymbol{\gamma} - \boldsymbol{\eta}'\mathbf{Q}_{11}^{-1}\boldsymbol{\eta}}\boldsymbol{\eta}'\mathbf{Q}_{11}^{-1}. \qquad \text{(A.5)}
\end{aligned}$$

Hence, combining (A.3), (A.4) and (A.5),

$$\mathbf{C}_{21}\mathbf{C}_{11}^{-1} = \mathbf{Q}_{21}\mathbf{Q}_{11}^{-1} + \left(\mathbf{Q}_{21}\mathbf{Q}_{11}^{-1}\boldsymbol{\eta} - \left(\mathbf{Q}_{21}\boldsymbol{\gamma}_1 + \mathbf{Q}_{22}\boldsymbol{\gamma}_2\right)\right)\frac{1}{\boldsymbol{\gamma}'\mathbf{Q}\boldsymbol{\gamma} - \boldsymbol{\eta}'\mathbf{Q}_{11}^{-1}\boldsymbol{\eta}}\boldsymbol{\eta}'\mathbf{Q}_{11}^{-1}. \qquad \text{(A.6)}$$

As

$$\begin{aligned}
\boldsymbol{\gamma}'\mathbf{Q}\boldsymbol{\gamma} &= \boldsymbol{\gamma}_1'\mathbf{Q}_{11}\boldsymbol{\gamma}_1 + 2\boldsymbol{\gamma}_1'\mathbf{Q}_{21}'\boldsymbol{\gamma}_2 + \boldsymbol{\gamma}_2'\mathbf{Q}_{22}\boldsymbol{\gamma}_2 \\
\boldsymbol{\eta}'\mathbf{Q}_{11}^{-1}\boldsymbol{\eta} &= \boldsymbol{\gamma}_1'\mathbf{Q}_{11}\boldsymbol{\gamma}_1 + 2\boldsymbol{\gamma}_1'\mathbf{Q}_{21}'\boldsymbol{\gamma}_2 + \boldsymbol{\gamma}_2'\mathbf{Q}_{21}\mathbf{Q}_{11}^{-1}\mathbf{Q}_{21}'\boldsymbol{\gamma}_2,
\end{aligned}$$

it follows that

$$\boldsymbol{\gamma}'\mathbf{Q}\boldsymbol{\gamma} - \boldsymbol{\eta}'\mathbf{Q}_{11}^{-1}\boldsymbol{\eta} = \boldsymbol{\gamma}_2'\left(\mathbf{Q}_{22} - \mathbf{Q}_{21}\mathbf{Q}_{11}^{-1}\mathbf{Q}_{21}'\right)\boldsymbol{\gamma}_2. \qquad \text{(A.7)}$$

Further,

$$\begin{aligned}
\mathbf{Q}_{11}^{-1}\boldsymbol{\eta} &= \boldsymbol{\gamma}_1 + \mathbf{Q}_{11}^{-1}\mathbf{Q}_{21}'\boldsymbol{\gamma}_2 \qquad \text{(A.8)} \\
\mathbf{Q}_{21}\mathbf{Q}_{11}^{-1}\boldsymbol{\eta} - \left(\mathbf{Q}_{21}\boldsymbol{\gamma}_1 + \mathbf{Q}_{22}\boldsymbol{\gamma}_2\right) &= \left(\mathbf{Q}_{21}\mathbf{Q}_{11}^{-1}\mathbf{Q}_{21}' - \mathbf{Q}_{22}\right)\boldsymbol{\gamma}_2. \qquad \text{(A.9)}
\end{aligned}$$

Combining (A.6), (A.7), (A.8) and (A.9), the result follows that

$$\mathbf{C}_{21}\mathbf{C}_{11}^{-1} = \mathbf{Q}_{21}\mathbf{Q}_{11}^{-1} - \left(\mathbf{Q}_{22} - \mathbf{Q}_{21}\mathbf{Q}_{11}^{-1}\mathbf{Q}_{21}'\right)\boldsymbol{\gamma}_2\frac{\boldsymbol{\gamma}_1' + \boldsymbol{\gamma}_2'\mathbf{Q}_{21}\mathbf{Q}_{11}^{-1}}{\boldsymbol{\gamma}_2'\left(\mathbf{Q}_{22} - \mathbf{Q}_{21}\mathbf{Q}_{11}^{-1}\mathbf{Q}_{21}'\right)\boldsymbol{\gamma}_2}.$$

A.2 Proof of Theorem 1

Under the stated assumptions,

$$\begin{aligned}\text{plim}\left(\widehat{\boldsymbol{\Gamma}}\right) &= \boldsymbol{\gamma}\beta + \boldsymbol{\alpha};\\ \text{plim}\left(\widehat{\boldsymbol{\gamma}}\right) &= \boldsymbol{\gamma}.\end{aligned}$$

Hence

$$\text{plim}\left(\widehat{\pi}_j\right) = \frac{\gamma_j \beta + \alpha_j}{\gamma_j} = \beta + \frac{\alpha_j}{\gamma_j},$$

for $j = 1, ..., L$. As $s < L/2$, more than 50% of the αs are equal to zero and hence it follows that more than 50% of the elements of $\text{plim}\left(\widehat{\boldsymbol{\pi}}\right)$ are equal to β. Using a continuity theorem, it then follows that

$$\text{plim}\left(\widehat{\beta}_m\right) = \text{median}\left\{\text{plim}\left(\widehat{\boldsymbol{\pi}}\right)\right\} = \beta.$$

For the limiting distribution, let $\boldsymbol{\delta}_1$ be the s-vector with elements

$$\delta_j = \frac{\alpha_j}{\gamma_j},$$

for $j = 1, ..., s$. Let $\boldsymbol{\delta} = \begin{pmatrix} \boldsymbol{\delta}_1' & \mathbf{0}_{L-s}' \end{pmatrix}'$. Partition $\widehat{\boldsymbol{\pi}}$ accordingly as $\widehat{\boldsymbol{\pi}} = \begin{pmatrix} \widehat{\boldsymbol{\pi}}_1' & \widehat{\boldsymbol{\pi}}_2' \end{pmatrix}'$. Under the standard conditions, the limiting distribution of $\widehat{\boldsymbol{\pi}}$ is given by

$$\sqrt{n}\left(\widehat{\boldsymbol{\pi}} - \left(\beta \boldsymbol{\iota}_L + \boldsymbol{\delta}\right)\right) \xrightarrow{d} N\left(0, \boldsymbol{\Sigma}_\pi\right).$$

As $\widehat{\beta}_m = \text{median}\left(\widehat{\boldsymbol{\pi}}\right)$,

$$\begin{aligned}\sqrt{n}\left(\widehat{\beta}_m - \beta\right) &= \sqrt{n}\left(\text{median}\left(\widehat{\boldsymbol{\pi}}\right) - \beta\right)\\ &= \text{median}\left(\sqrt{n}\left(\widehat{\boldsymbol{\pi}} - \beta \boldsymbol{\iota}_L\right)\right).\end{aligned}$$

As

$$\sqrt{n}\left(\widehat{\boldsymbol{\pi}} - \beta \boldsymbol{\iota}_L\right) = \begin{pmatrix} \sqrt{n}\left(\widehat{\boldsymbol{\pi}}_1 - \left(\beta \boldsymbol{\iota}_s + \boldsymbol{\delta}_1\right)\right) + \sqrt{n}\boldsymbol{\delta}_1 \\ \sqrt{n}\left(\widehat{\boldsymbol{\pi}}_2 - \beta \boldsymbol{\iota}_{L-s}\right) \end{pmatrix},$$

it follows that

$$\sqrt{n}\left(\widehat{\beta}_m - \beta\right) = \text{median}\left(\sqrt{n}\left(\widehat{\boldsymbol{\pi}} - \beta \boldsymbol{\iota}_L\right)\right) \xrightarrow{d} q_{[l],L-s}.$$

B Additional Simulation Results

B.1 Equal Strength Instruments

Figures B1a and B1b illustrate the different behaviours of the Lasso and post-Lasso estimators for the design as in Table 1. Figure B1a shows the bias and standard deviations of the two estimators for different values of λ/n for $n = 2000$, again from 1000 replications. It is clear that the Lasso estimator exhibits a positive bias for all values of λ, declining from that of the naive 2SLS estimator to the minimum bias of 0.0664 at $\lambda/n = 0.0060$. In contrast, the post-Lasso estimator is (much) less biased, obtaining its minimum bias of 0.0068 at the value of λ/n of 0.0965. Figure B1b displays the same information but now as a function of the LARS steps, where additional variables enter the model (we have omitted 3 replications where there were Lasso steps). At step 3, the correct 3 invalid instruments have been selected 991 times out of the 997 replications, and the post-Lasso estimator has a bias there of 0.0058, only fractionally larger than that of the oracle 2SLS estimator. In contrast, the Lasso estimator for β still has a substantial upward bias at step 3. Its bias decreases from 0.116 at step 3 to a minimum of 0.0650 at step 8. The bias of the post-Lasso estimator increases again after step 3, reaching the same bias as the Lasso estimator at the last step, as there $\lambda_n = 0$ and the Lasso and post-Lasso estimators are equal.

As the results in Table 1 show, the difference in bias between the Lasso and post-Lasso estimators is larger for the *cvse* procedure compared to the *cv* procedure, as the *cvse* procedure selects a larger value for λ_n and hence shrinks the α coefficients more towards 0. This results in a larger bias in the estimator for β, as depicted in Figure B1a.

Table B1 presents results for the median and Adaptive Lasso estimators. The estimation results for the adaptive Lasso are based on setting $v = 1$. The resulting estimators are denoted "ALasso". As L is even here, the median is defined as $\widehat{\beta}_m = \left(\widehat{\pi}_{[5]} + \widehat{\pi}_{[6]}\right)/2$, where $\widehat{\pi}_{[j]}$ is the j-th order statistic. The results show the oracle properties of the adaptive Lasso procedure, especially for the post-ALasso$_{cvse}$ estimator, with its estimation results very close to that of the oracle 2SLS estimator for $n = 2000$ and $n = 10,000$.

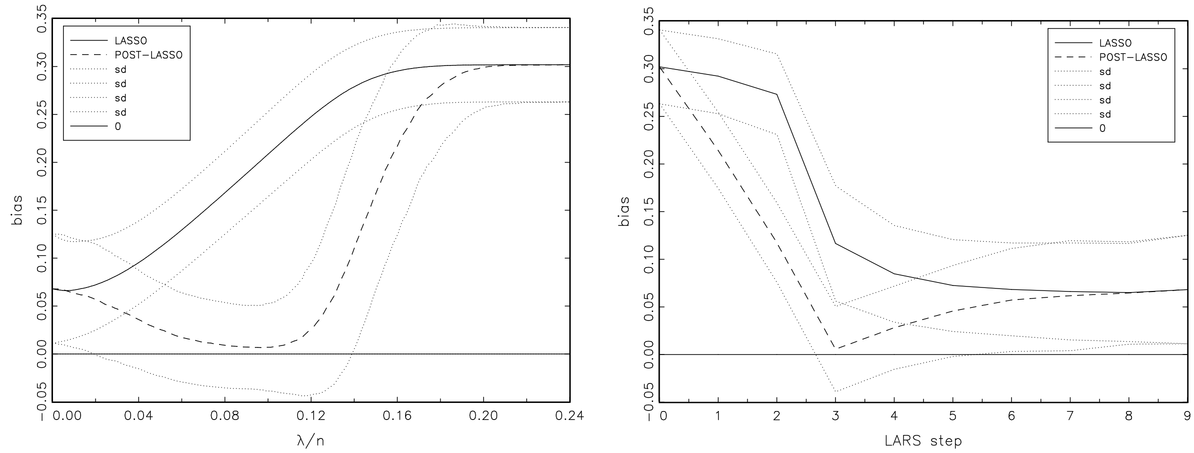

Figures B1a and B1b. Bias and standard deviations of Lasso and post-Lasso estimators as functions of λ/n, and LARS steps. Same design as in Table 1, $n = 2000$. 3 replications out of 1000 omitted in Figure B1b due to Lasso steps.

The design in Tables 1 and B1 has $L = 10$, $s = 3$ and $\delta_1 = \delta_2 = \delta_3 > 0$. As the median is defined as $\widehat{\beta}_m = \left(\widehat{\pi}_{[5]} + \widehat{\pi}_{[6]}\right)/2$, it follows that

$$\sqrt{n}\left(\widehat{\beta}_m - \beta\right) = \text{median}\left(\sqrt{n}\left(\widehat{\boldsymbol{\pi}} - \beta\boldsymbol{\iota}_{10}\right)\right) \xrightarrow{d} q_{[5,6],7}$$

where $q_{[5,6],7}$ is the average of the fifth and sixth order statistic of the limiting distribution

$$\sqrt{n}\left(\begin{pmatrix} \widehat{\pi}_4 \\ \vdots \\ \widehat{\pi}_{10} \end{pmatrix} - \beta\boldsymbol{\iota}_7\right) \xrightarrow{d} N\left(0, \boldsymbol{\Sigma}_\pi^*\right).$$

For the design in Table B1, $\boldsymbol{\Sigma}_\pi^* = 25\mathbf{I}_7$, as $\sigma_\varepsilon^2 = 1$ and $1/\gamma_j^2 = 25$ for $j = 4, ..., 10$. From a simple simulation, drawing repeatedly from the $N\left(0, 25\mathbf{I}_7\right)$ distribution, we find that $E\left[q_{[5,6],7}\right] = 2.78$. Therefore $E\left[q_{[5,6],7}\right]/\sqrt{n} = 0.0278$ for $n = 10,000$, almost exactly the result found for the bias of $\widehat{\beta}_m$ in Table B1. For this design, the asymptotic bias of the median estimator is affected by the number of invalid instruments in the following way. For $n = 10,000$ we get for $s = 4$, 2, 1, 0 respectively $E\left[q_{[5,6],6}\right]/\sqrt{n} = 0.0477$; $E\left[q_{[5,6],8}\right]/\sqrt{n} = 0.0156$; $E\left[q_{[5,6],9}\right]/\sqrt{n} = 0.0069$; and $E\left[q_{[5,6],10}\right]/\sqrt{n} = 0$.

Table B1. Estimation results for Adaptive Lasso estimators for β; $L = 10$, $s = 3$, $\widetilde{\gamma}_1 = \widetilde{\gamma}_2$

β	bias	std dev	rmse	mad	av. # instr selected as invalid [min, max]	freq. all invalid instr selected
$n = 500$						
$\widehat{\beta}_m$	0.1197	0.1029	0.1578	0.1159		
ALasso$_{cv}$	0.0952	0.0981	0.1366	0.0934	4.62 [2,9]	0.974
Post-ALasso$_{cv}$	0.0732	0.1182	0.1390	0.0894		
ALasso$_{cvse}$	0.1984	0.0847	0.2157	0.1974	2.63 [0,6]	0.583
Post-ALasso$_{cvse}$	0.0703	0.1156	0.1353	0.0859		
$n = 2000$						
$\widehat{\beta}_m$	0.0634	0.0502	0.0809	0.0648		
ALasso$_{cv}$	0.0350	0.0496	0.0607	0.0403	4.17 [3,9]	1
Post-ALasso$_{cv}$	0.0281	0.0573	0.0638	0.0386		
ALasso$_{cvse}$	0.0960	0.0434	0.1053	0.0977	3.03 [3,5]	1
Post-ALasso$_{cvse}$	0.0059	0.0433	0.0437	0.0287		
$n = 10,000$						
$\widehat{\beta}_m$	0.0277	0.0225	0.0357	0.0278	3	1
ALasso$_{cv}$	0.0111	0.0224	0.0250	0.0169	3.85 [3,9]	1
Post-ALasso$_{cv}$	0.0081	0.0250	0.0263	0.0169		
ALasso$_{cvse}$	0.0382	0.0191	0.0428	0.0391	3.01 [3,4]	1
Post-ALasso$_{cvse}$	0.0008	0.0184	0.0184	0.0127		

Notes: Results from 1000 MC replications; $\beta = 0$; $\rho = 0.25$; $a = 0.2$; $\widetilde{\gamma}_2$= 0.2

Having all elements of $\boldsymbol{\delta}_1$ with the same sign is clearly the worst case scenario for the asymptotic bias of the median estimator. The best case scenario is for even s, if half the elements in $\boldsymbol{\delta}_1$ are positive and half negative, as we then have that $\sqrt{n}\left(\widehat{\beta}_m - \beta\right)$ converges to the median of the limiting distribution of $\sqrt{n}\left(\widehat{\boldsymbol{\pi}}_2 - \beta\ \boldsymbol{\iota}_{L-s}\right)$, and therefore has no asymptotic bias.

For the results in Table B1, for $n = 2000$, the means of the estimates $\widehat{\alpha}_{m,j}$ for the positive $\alpha_j = 0.2$, $j = 1, .., 3$, are approximately 0.187, whereas the means of the estimates for the $\alpha_j = 0$, $j = 4, ..., 10$, are approximately 0.0186. For $n = 10,000$, these are approximately 0.194 and 0.0085. The ratios of the biases for $n = 10,000$, relative to those of $n = 2000$ are approximately 0.45 which is equal to $\sqrt{2000}/\sqrt{10,000}$, confirming that the bias in $\widehat{\boldsymbol{\alpha}}_m$ decreases at the $\sqrt{n}$ rate.

B.2 Information content

We distinguish two different measures of information in the IV Lasso selection problem. First, as mentioned in Section 5, the information content for the estimation of β in the oracle instrumental variables model is characterised by the concentration parameter μ_n^2, Rothenberg (1984), which is here given by

$$\mu_n^2 = \frac{\boldsymbol{\gamma}_2' \mathbf{Z}_2' \mathbf{M}_{Z_1} \mathbf{Z}_2 \boldsymbol{\gamma}_2}{\sigma_v^2}.$$

μ_n^2 is approximately equal to $n\,(L - s)\,\widetilde{\gamma}_2^2 = 140$ for the $n = 500$ cases above, which is equivalent to a first-stage population F-test value for the null $H_0 : \boldsymbol{\gamma}_2 = 0$ of $n\widetilde{\gamma}_2^2 = 20$. This is a reasonably large value of the F-test, see Staiger and Stock (1997) and Stock and Yogo (2005), which is reflected in the good properties of the oracle 2SLS estimator. Clearly, though, the Lasso procedures do not perform well for the $n = 500$ case, especially when $\widetilde{\gamma}_1 = 3\widetilde{\gamma}_2$.

The weak instrument asymptotics of Staiger and Stock (1997) is obtained by considering $\boldsymbol{\gamma}_2$ in a neighbourhood of 0, as $\boldsymbol{\gamma}_2 = \mathbf{c}_{\gamma_2}/\sqrt{n}$, with $\mathbf{c}_{\gamma_2}$ a vector of constants. The information content μ_n^2 does then not increase with the sample size and converges to $\mu_c^2 = \mathbf{c}_{\gamma_2}' \widetilde{Q}_{22} \mathbf{c}_{\gamma_2} / \sigma_v^2$ as $n \to \infty$. The oracle 2SLS estimator is in that case not consistent and converges to a random variable with expected value different from β, with the difference larger for smaller values of μ_c^2.

Second, a measure of information for the Lasso selection is the (squared) Signal to Noise Ratio (SNR), see e.g. Bühlmann and Van de Geer (2011, p. 25), defined as

$$\eta^2 = \frac{\boldsymbol{\alpha}' \mathbf{C} \boldsymbol{\alpha}}{\sigma_\varepsilon^2} = \frac{\boldsymbol{\alpha}_1' \mathbf{C}_{11} \boldsymbol{\alpha}_1}{\sigma_\varepsilon^2},$$

where, as before, $\mathbf{C} = \text{plim}\left(\frac{1}{n}\widetilde{\mathbf{Z}}'\widetilde{\mathbf{Z}}\right)$ and $\mathbf{C}_{11} = \text{plim}\left(\frac{1}{n}\widetilde{\mathbf{Z}}_1'\widetilde{\mathbf{Z}}_1\right)$. From the proof of Proposition 1 we obtain

$$\boldsymbol{\alpha}_1' \mathbf{C}_{11} \boldsymbol{\alpha}_1 = \boldsymbol{\alpha}_1' \mathbf{Q}_{11} \boldsymbol{\alpha}_1 - \frac{\left(\boldsymbol{\gamma}_1' \mathbf{Q}_{11} \boldsymbol{\alpha}_1 + \boldsymbol{\gamma}_2' \mathbf{Q}_{21} \boldsymbol{\alpha}_1\right)^2}{\boldsymbol{\gamma}_1' \mathbf{Q}_{11} \boldsymbol{\gamma}_1 + 2\, \boldsymbol{\gamma}_2' \mathbf{Q}_{21} \boldsymbol{\gamma}_1 + \boldsymbol{\gamma}_2' \mathbf{Q}_{22} \boldsymbol{\gamma}_2}. \quad \text{(B.1)}$$

It follows from (B.1) that if we multiply $\boldsymbol{\alpha}_1$ by a factor m, η^2 gets multiplied by m^2 whereas multiplying $\boldsymbol{\gamma}$ by a factor m does not affect the value of η^2.

For the Monte Carlo design in Section 5, with $\sigma_\varepsilon^2 = 1$, it follows that

$$\begin{aligned}\eta^2 &= sa^2 - \frac{(sa\widetilde{\gamma}_1)^2}{s\widetilde{\gamma}_1^2 + (L-s)\widetilde{\gamma}_2^2} \\ &= \frac{(L-s)\,a^2}{\left(\frac{\widetilde{\gamma}_1}{\widetilde{\gamma}_2}\right)^2 + \frac{L-s}{s}},\end{aligned}$$

and so the SNR is here directly influenced by the relative value $|\widetilde{\gamma}_1/\widetilde{\gamma}_2|$, with an increase in this value decreasing the value of η^2. For the unequal strength design above, $\eta^2 = 0.0247$, and for the equal strength design it is $\eta^2 = 0.0840$. As Zou (2006) indicated, the smaller value of the SNR in the unequal design explains the poorer performance of the adaptive Lasso estimators for a given sample size, as depicted in Figures 1b and 1c.

In order to illustrate the performance of the adaptive Lasso estimator in relation to the value of η^2, Table B2 shows the rejection frequencies at the 10% level of the robust Wald tests $W_{\beta,r}$ based on the oracle and post-ALasso$_{ah}$ estimators, for the $n = 500$ unequal strength case as in Section 5.1, but increasing $\boldsymbol{\alpha}$ by a multiplicative factor $m = \sqrt{1}, \sqrt{3}, ..., \sqrt{9}$, and hence increasing η^2 by a multiplicative factor m^2. At $m^2 = 9$, the post-ALasso$_{ah}$ estimator again behaves like the oracle estimator. Increasing the value of a, whilst keeping $\boldsymbol{\gamma}$ constant, increases of course the bias of the naive 2SLS estimator as is clear from (23). The value for $n\eta^2$, which is akin to a population Wald statistic for testing $H_0 : \boldsymbol{\alpha}_1 = 0$, ranges from 12.35 for $m = 1$ to 111.15 for $m = 3$.

Increasing $\widetilde{\gamma}_1$ and $\widetilde{\gamma}_2$ by the same multiplicative factor whilst keeping a constant does not alter η^2, and does not lead to an improvement of the performance of the Wald test $W_{\beta,r}$ when keeping the sample size constant, as confirmed by the results in Table B2. The bias of the naive 2SLS estimator decreases here with increasing values of m.

Table B2. Rejection frequencies of $W_{\beta,r}$ at 10% level, varying $\boldsymbol{\alpha}$ or $\boldsymbol{\gamma}$

	m^2				
$\boldsymbol{\alpha} \times m$	1	3	5	7	9
post-ALasso$_{ah}$	0.9300	0.3130	0.1350	0.1100	0.1070
Oracle	0.1020	0.1030	0.1020	0.1010	0.1040
η^2	0.0247	0.0741	0.1235	0.1729	0.2223
$\boldsymbol{\gamma} \times m$					
post-ALasso$_{ah}$	0.9300	0.9270	0.9340	0.9360	0.9360
Oracle	0.1020	0.0960	0.0980	0.1010	0.0980
η^2	0.0247	0.0247	0.0247	0.0247	0.0247

Notes: $n = 500$, same design as in Table 2 when $m = 1$, 1000 MC replications

Multiplying both $\boldsymbol{\gamma}$ and $\boldsymbol{\alpha}$ by a factor m increases both μ_n^2 and η^2 by a factor m^2. Table B3 displays estimation results for the unequal strength design for $n = 500$, multiplying a, $\widetilde{\gamma}_1$ and $\widetilde{\gamma}_2$ by $m = \sqrt{20}$. The estimation results are very similar to those for $n = 10,000$ as reported in Tables 2 and 3, and so the information increase due to multiplying the parameters by m is here very similar to multiplying the sample size by a factor m^2.

Table B3. Estimation results for β; $n = 500$, $\widetilde{\gamma}_1 = 3\widetilde{\gamma}_2$, $a = \widetilde{\gamma}_2 = 0.8944$

β	bias	std dev	rmse	mad	av. # instr selected as invalid [min, max]	freq. all s invalid instr selected
2SLS	0.2650	0.0106	0.2652	0.2651	0	0
2SLS or	-0.0002	0.0191	0.0191	0.0128	3	1
$\widehat{\beta}_m$	0.0272	0.0229	0.0356	0.0267		
Post-ALasso$_{cvse}$	0.0003	0.0194	0.0194	0.0132	3.02 [3,5]	1
Post-ALasso$_{ah}$	0.0000	0.0192	0.0192	0.0130	3.02 [3,5]	1

Notes: Results from 1000 MC replications; $L = 10$, $s = 3$, $\beta = 0$, $\rho = 0.25$

The values of η^2 or $n\eta^2$ do not convey the same information about the performance of the adaptive Lasso estimators as μ_n^2 does for the oracle 2SLS estimator. We first illustrate this with an example where the coefficients in $\boldsymbol{\alpha}_1$ take different values. In this case, the performance of the Lasso estimator is driven by how well it does in selecting the variable with the smallest value $|\alpha_j|$, $j = 1, ..., s$. If we for example change $\boldsymbol{\alpha}_1$ to $\boldsymbol{\alpha}_1 = \begin{pmatrix} 0.1 & 0.2 & 0.2063 \end{pmatrix}'$, we get the same value of $\eta^2 = 0.0247$ in the unequal strength design, but as Figure B2 shows, a much larger sample size is needed for the inference based on the post-ALasso$_{cvse}$ and post-ALasso$_{ah}$ estimators to be similar to that of the oracle estimator, due to the presence of the smaller coefficient $\alpha_1 = 0.1$.

For the oracle properties of the adaptive Lasso estimator, we need that $|\boldsymbol{\alpha}_1|_{\min} \gg O\left(n^{-1/2}\right)$. Similar to μ_n^2, the population Wald statistic $n\eta^2$ does not increase with the sample size if $\boldsymbol{\alpha}_1 = \mathbf{c}_{\alpha_1}/\sqrt{n}$. Although the naive 2SLS estimator is then consistent in this finite s setting, provided $\boldsymbol{\gamma}_2 \gg O\left(n^{-1/2}\right)$, the adaptive Lasso estimator will not have oracle properties in this case, and the properties of the Wald test $W_{\beta,r}$ do not improve with an increasing sample size, as the information does not increase and $\widehat{\beta}_{ad}^{(n)}$ has an asymptotic bias. For example, setting $\boldsymbol{\alpha}_1 = a_n \boldsymbol{\iota}_s$ in the equal strength instruments design, with $a_n = 0.2 * \sqrt{500}/\sqrt{n}$, gives rejection frequencies of the robust Wald test based on the post-ALasso$_{ah}$ estimator at the 10% level of 35%, 33% and 34% for $n = 500$,

$n = 2000$ and $n = 10,000$ respectively. For the unequal strength instruments example above, setting $\boldsymbol{\alpha}_1 = \begin{pmatrix} a_n & 0.2 & 0.2063 \end{pmatrix}$ with $a_n = 0.1 * \sqrt{500}/\sqrt{n}$, we get rejection frequencies of the post-ALasso$_{ah}$ based Wald test of 56%, 53% and 54% respectively at $n = 2000$, $n = 10,000$ and $n = 30,000$. In both examples, the adaptive Lasso procedure does not select the full set of invalid instruments as invalid in large samples. This leads to an asymptotic bias, as the random variable $\sqrt{n}\left(\widehat{\beta}_{ad}^{(n)} - \beta\right)$ then does not have a mean of zero when $n \to \infty$, as $a_n = O\left(n^{-1/2}\right)$. Of course, in the finite dimension, fixed parameters case we consider here, we have the limiting distribution of the adaptive Lasso estimator as in (21), but, as shown in Figure B2, the presence of small coefficients in $\boldsymbol{\alpha}_1$ may affect the behaviour of the estimator in any given application.

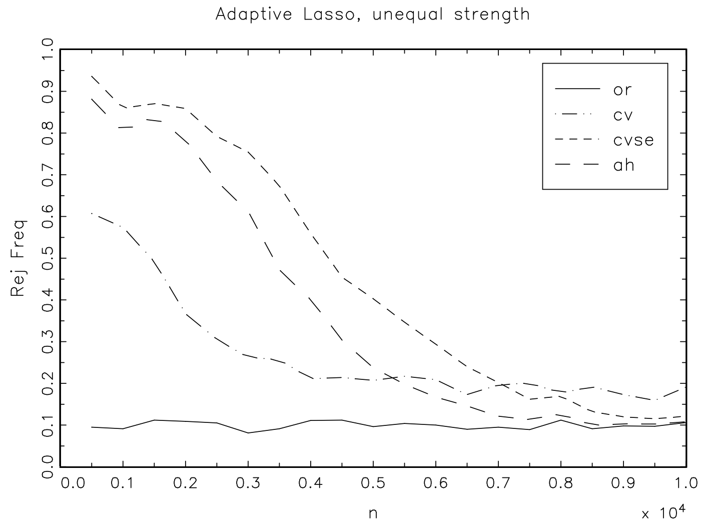

Figure B2. Rejection frequencies of robust Wald tests for H_0: $\beta = 0$ at 10% level as a function of sample size, in steps of 500. Unequal strength instruments design, Post-ALasso, $\alpha_1 = \begin{pmatrix} 0.1 & 0.2 & 0.2063 \end{pmatrix}$. Based on 1000 MC replications for each sample size.

The Sargan test based on the naive 2SLS estimator is related to the SNR, as it is a score test for $H_0 : \boldsymbol{\alpha}_C = 0$ in the model

$$\mathbf{y} = \mathbf{d}\beta + \mathbf{Z}_C\boldsymbol{\alpha}_C + \boldsymbol{\varepsilon},$$

where $\mathbf{Z}_C$ is any $(L-1)$ subset of instruments from $\mathbf{Z}$. The SNR η^2 is not affected when $\boldsymbol{\gamma}$ is multiplied by a factor m and therefore is not affected by weak instruments, where the value of $\boldsymbol{\gamma}$ is such that the concentration parameter μ_n^2 is small. However, it

is well known that weak instruments decrease the power of the Sargan/Hansen test, see Staiger and Stock (1997) and Kitamura (2006), which would then affect the behaviour of the post-ALasso$_{ah}$ estimator, as the decrease in power will result in less instruments selected as invalid. This is illustrated in Table B4, which presents results for the unequal instrument, large a design as in Table B2, for $n = 500$, $a = 0.6$, $\widetilde{\gamma}_1 = 0.6$, $\widetilde{\gamma}_2 = 0.2$, and $\boldsymbol{\gamma}$ multiplied by a factor $1/m$ for $m^2 = 1, 3, ..., 9$. We present results here for the median bias, as the variability of the estimators increases substantially for larger values of m. For increasing value of m, the number of selected invalid instruments decreases for the ALasso$_{ah}$ estimator, whilst that of the ALasso$_{cvse}$ actually increases, and the median bias of the ALasso$_{ah}$ estimator increases relative to that of the ALasso$_{cvse}$ estimator. Increasing the p-value for the Hansen test decreases the bias of the ALasso$_{ah}$ estimator. For example, for $m = 3$, setting the p-value to 0.2 instead of 0.016 increases the average number of selected instruments as invalid to 3.13 and reduces the median bias of the estimator to 0.1022. The post-ALasso$_{ah}$ estimator is, however, a much noisier estimator here with a standard deviation of 0.66 compared to 0.35 for the post-ALasso$_{cvse}$ estimator and 0.21 for the oracle 2SLS estimator.

Table B4. Median bias for 2SLS estimators of β; $L = 10$, $s = 3$

	m^2				
$\boldsymbol{\gamma} \times (1/m)$	1	3	5	7	9
2SLS	0.7883	1.3311	1.6778	1.9464	2.1570
2SLS or	0.0087	0.0344	0.0504	0.0666	0.0740
post-ALasso$_{cvse}$	0.0138	0.0410	0.0632	0.0899	0.0992
# Inv Inst	3.07	3.10	3.17	3.26	3.31
post-ALasso$_{ah}$	0.0103	0.0348	0.0556	0.0920	0.1257
# Inv Inst	3.01	3.01	2.88	2.61	2.37
μ_n^2	678.51	225.66	136.40	96.86	75.14

Notes: $n = 500$, $a = 0.6$, $\widetilde{\gamma}_1 = 3\widetilde{\gamma}_2$, $\widetilde{\gamma}_2 = 0.2/m$, 1000 MC replications

For another example where the same value of η^2 does not imply the same finite sample behaviour even when all the coefficients in $\boldsymbol{\alpha}_1$ are the same, we consider a design with correlated instruments. Figures B3a-B3c below show the rejection frequencies of the robust Wald test $W_{\beta,r}$ as in Figures 1a-1c for equal and unequal strength instruments, but where the instruments are correlated and distributed as follows

$$\mathbf{Z}_{i.} \sim N(0, \boldsymbol{\Sigma}),$$

with the elements of $\boldsymbol{\Sigma}$ given by

$$\boldsymbol{\Sigma}_{r,k} = \omega^{|r-k|}$$

and $\omega = 0.5$.

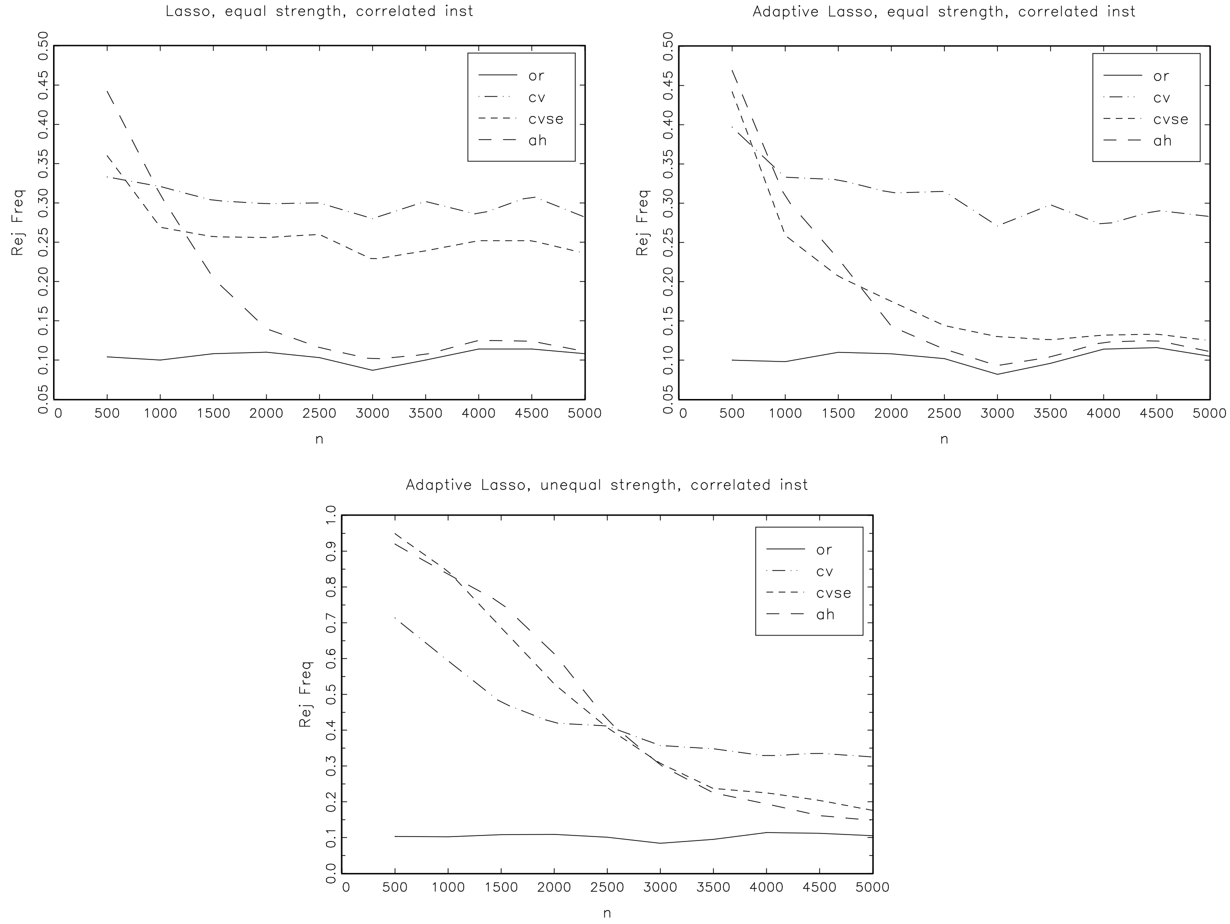

Figures B3a, B3b and B3c. Rejection frequencies of robust Wald tests for H_0: $\beta = 0$ at 10% level as a function of sample size, in steps of 500. Correlated instruments, equal strength instruments design, Post-Lasso in Figure B3a, Post-ALasso in Figure 3Bb. Unequal strength instruments design, Post-ALasso in Figure 3Bc. Based on 1000 MC replications for each sample size.

As in the designs of Figures 1a-1c, $\boldsymbol{\alpha}_1 = a\boldsymbol{\iota}_s$, $\boldsymbol{\gamma}_1 = \widetilde{\gamma}_1\boldsymbol{\iota}_s$, $\boldsymbol{\gamma}_2 = \widetilde{\gamma}_2\boldsymbol{\iota}_{L-s}$, $L = 10$, $s = 3$ and for the unequal strength design $\widetilde{\gamma}_1 = 3\widetilde{\gamma}_2$. We set the parameters a and $\widetilde{\gamma}_2$ such that $\mu^2 = \text{plim}\,(\mu_n^2/n)$ and η^2 are equal to those of the designs for Figures 1a-1c. This results in $\widetilde{\gamma}_2 = 0.1321$ for both designs, $a = 0.1552$ in the equal instruments design, and

$a = 0.1448$ in the unequal instruments design. As the figures show, a larger sample size n is needed here for a performance of the Wald test $W_{\beta,r}$ similar to that of the uncorrelated instruments designs.

Assumption 4 states that the instruments are relevant in the sense that $\gamma_j \neq 0$, for $j = 1, ..., L$. If this assumption is relaxed and some of the γ_j are equal to 0, then the instruments are potentially invalid for two reasons as the relevance and/or exclusion condition may fail. If $\gamma_j = 0$, then $|\pi_j| = \left|\left(\beta\gamma_j + \alpha_j\right)/\gamma_j\right|$ is either ∞ for instruments with $\alpha_j \neq 0$, or ill defined as $\beta + 0/0$. The median and adaptive Lasso estimators still have the properties as stated in Section 4 if we make the assumption that more than 50% of the instruments are valid *and* relevant. Another remedy to a relevance problem would be to do an initial selection of the instruments by for example a Lasso selection in the model $\mathbf{d} = \mathbf{Z}\boldsymbol{\gamma} + \mathbf{v}$, with the identification condition then that less than 50% of the selected (i.e. sufficiently strong) instruments are invalid. This is the approach taken by Guo et al. (2018), who then proceed to select the invalid instruments by a pairwise comparison of the $\widehat{\pi}_j$.

References

Abadir, K.M., and J.R. Magnus, (2005), *Matrix Algebra* (Econometric Exercises), Cambridge University Press, Cambridge.

Kitamura, Y. (2006), Specification Tests with Instrumental Variables and Rank Deficiency. In D. Corbae, S. Durlauf, & B. Hansen (Eds.), *Econometric Theory and Practice: Frontiers of Analysis and Applied Research,* 59-81. Cambridge: Cambridge University Press.
