# Supplementary material for: On the Use of the Lasso for Instrumental Variables Estimation with Some Invalid Instruments
Source: J Am Stat Assoc. 2018 Nov 13;114(527):1339–50. doi: 10.1080/01621459.2018.1498346 (PMC6817329; doi:10.1080/01621459.2018.1498346)
Supplement: Supplemental Material [file UASA_A_1498346_SM7564.zip › UASA_A_1498346_Supplement/uk_biobank_data_cleaning_nmd_170703.docx]

# How to get access to Biobank data

We cannot directly disseminate individual level participant data because of our terms of access to the data. However, all researchers can access the UK Biobank data by applying at (<http://www.ukbiobank.ac.uk/register-apply/)>. The application procedure is described in detail on the website. We requested data on all individuals within the study, but the analysis is restricted to individuals with genotype data in the initial release of the UK Biobank data. If you need any further information about accessing the data please contact Neil Davies (neil.davies@bristol.ac.uk).

We cleaned the data using the Stata script below.

# Variables used in the empirical example

| Variable | ID |
| --- | --- |
| Systolic blood pressure | 4080 |
| Diastolic blood pressure | 4079 |
| Body mass index | 21001 |
| Year of birth | 34 |
| Self-reported sex | 31 |
| 15 genetic principal components | 22009 |
| Genotyping batch | 22000 |

# Scripts used to clean the Biobank data

We used the following script to clean the raw phenotypic data.

//Neil Davies 29/09/15

//This creates the data file for the paper. It contains all the BMI SNPs and the blood pressure outcomes.

cd "/Volumes/Height_BMI_and_schooling/UK biobank"

use "raw_data/Biobank_phenotypes_NMD_150417.dta", clear

//The BMI variants were extracted from the imputed data from the UK Biobank initial release using QCTOOL. The variants were recoded in additive dosage format using R.

joinby n_eid using "working data/bmi_SNPs_dosage",unmatched(both)

drop _m

//Blood pressure

egen out_sys_bp=rowmean(n_4080_0_1 n_4080_0_0)

egen out_dia_bp=rowmean(n_4079_0_1 n_4079_0_0)

//Anthropometry

gen out_bmi=n_21001_0_0

//Calculate age

gen cov_age=2008-n_34_0_0

egen cov_z_age=std(cov_age)

gen cov_z_age2=cov_z_age^2

gen cov_birth_year=n_34_0_0

gen cov_birth_year2=n_34_0_0^2

gen cov_birth_year3=n_34_0_0^3

//Generate male

gen cov_male=n_31_0_0

//Match in the additional genetic data

//These were calculated by the UK Biobank and were not manipulated.

joinby n_eid using "raw_data/genotype_PCA_data.dta",unmatched(both)

drop _m

//Restrict to the recommended Biobank sample:

//This was taken from the summary data provided with genome-wide

rename n_eid id_1

joinby id_1 using "raw_data/impv1.sample.dta",unmatched(both)

//drop all 350407 non-genotyped samples:

drop if _m!=3

//drop 31963 non-white individuals

drop if n_22006_0_0!=1

//drop 7948 related individuals

drop if n_22011_0_0!=.

//This leaves 112,338 individuals which is the number reported in the Biobank imputation example

//replace missing outcome with NA:

rename id_1 n_eid

drop _m

rename rs6091540_CTRUE rs6091540_CT

rename rs2836754_TRUEC rs2836754_TC

keep n_eid out_sys_bp-cov_birth_year3 cov_batch n_22009_0_1-n_22009_0_15 rs*

saveold "working data/biobank_bmi_bp_windmeijer_nmd_151119",replace
